# Acetylcholinesterase Inhibition in Rats and Humans Following Acute Fenitrothion Exposure Predicted by Physiologically Based Kinetic Modeling-Facilitated Quantitative *In Vitro* to *In Vivo* Extrapolation

**DOI:** 10.1021/acs.est.3c07077

**Published:** 2023-11-27

**Authors:** Jiaqi Chen, Shensheng Zhao, Sebastiaan Wesseling, Nynke I. Kramer, Ivonne M.C.M. Rietjens, Hans Bouwmeester

**Affiliations:** Division of Toxicology, Wageningen University and Research, Stippeneng 4, Wageningen 6708 WE, The Netherlands

**Keywords:** organophosphate pesticide, fenitrothion, acetylcholinesterase inhibition, physiologically based kinetic (PBK) model, quantitative *in vitro* to *in vivo* extrapolation (QIVIVE)

## Abstract

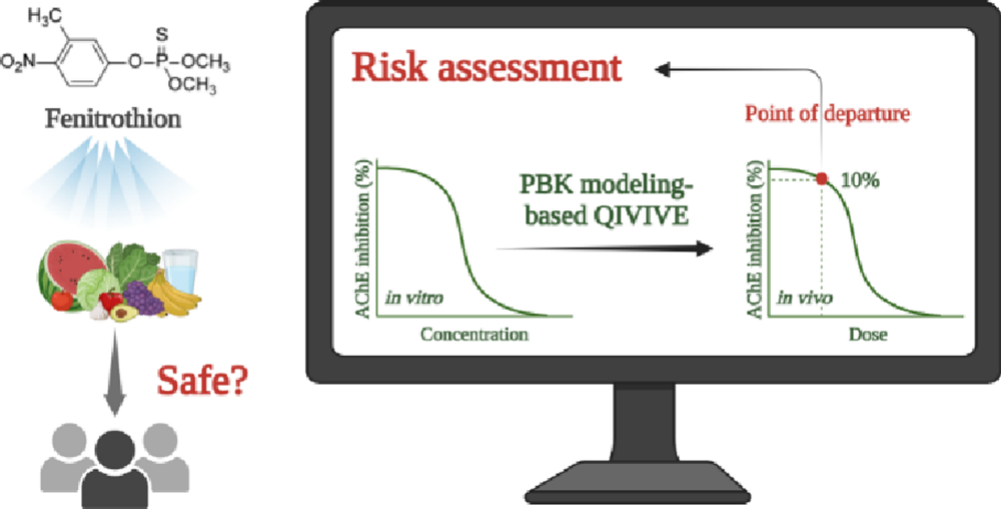

Worldwide use of organophosphate pesticides as agricultural chemicals aims to maintain a stable food supply, while their toxicity remains a major public health concern. A common mechanism of acute neurotoxicity following organophosphate pesticide exposure is the inhibition of acetylcholinesterase (AChE). To support Next Generation Risk Assessment for public health upon acute neurotoxicity induced by organophosphate pesticides, physiologically based kinetic (PBK) modeling-facilitated quantitative *in vitro* to *in vivo* extrapolation (QIVIVE) approach was employed in this study, with fenitrothion (FNT) as an exemplary organophosphate pesticide. Rat and human PBK models were parametrized with data derived from *in silico* predictions and *in vitro* incubations. Then, PBK model-based QIVIVE was performed to convert species-specific concentration-dependent AChE inhibition obtained from *in vitro* blood assays to corresponding *in vivo* dose–response curves, from which points of departure (PODs) were derived. The obtained values for rats and humans were comparable with reported no-observed-adverse-effect levels (NOAELs). Humans were found to be more susceptible than rats toward erythrocyte AChE inhibition induced by acute FNT exposure due to interspecies differences in toxicokinetics and toxicodynamics. The described approach adequately predicts toxicokinetics and acute toxicity of FNT, providing a proof-of-principle for applying this approach in a 3R-based chemical risk assessment paradigm.

## Abstract 2

An integration of PBK modeling-facilitated QIVIVE approach and *in vitro* blood AChE inhibition assay provides a 3R-based paradigm for human health risk assessment for organophosphate pesticide exposures.

## 1 Introduction

**Figure 1:**
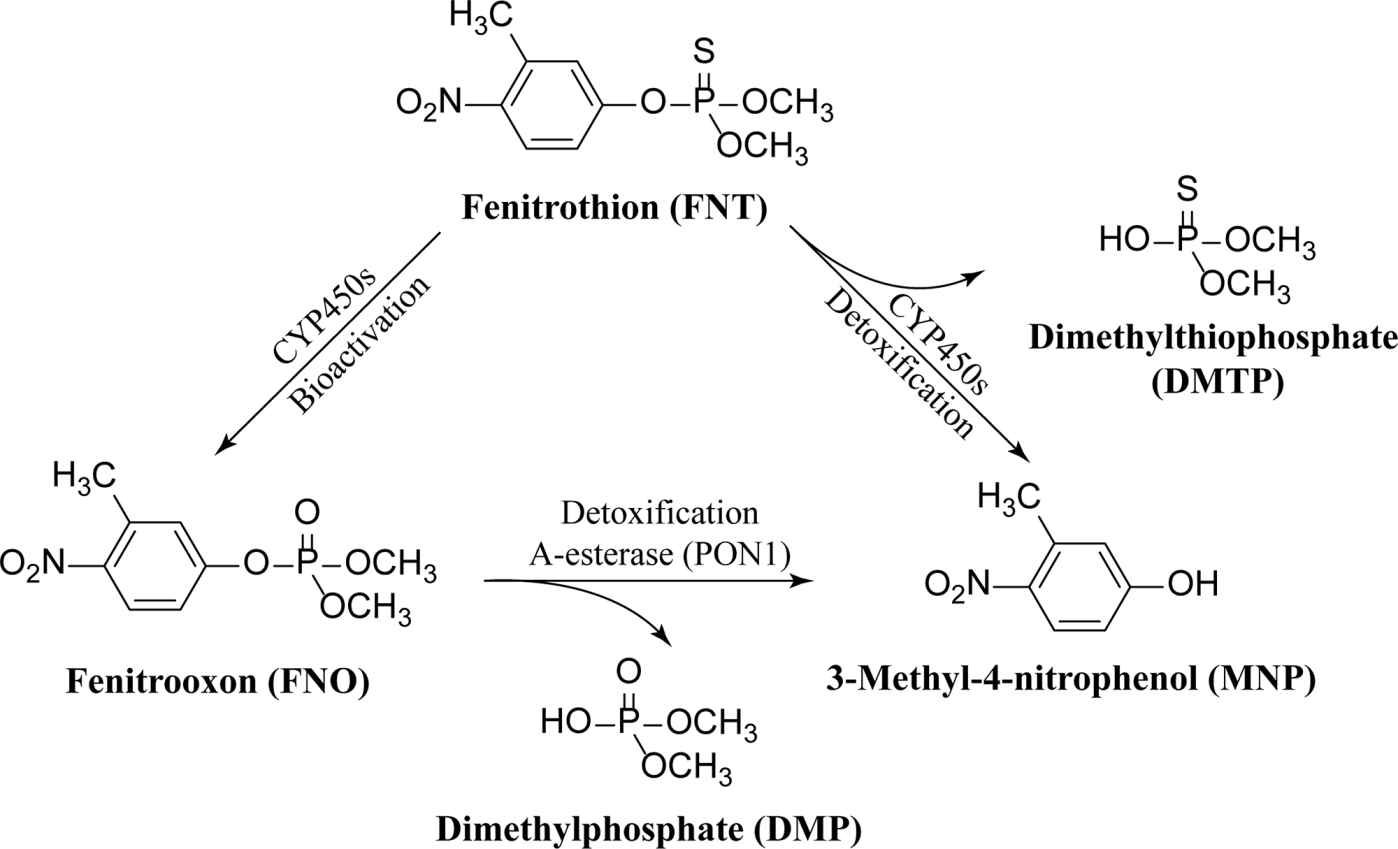
Major biotransformation pathways of fenitrothion (FNT) catalyzed by cytochromes P450 (CYP450s) and paraoxonase 1 (PON1) in rats and humans.^1,2^

The organophosphate pesticide fenitrothion (FNT, *O*,*O*-dimethyl *O*-4-nitro-*m*-tolyl phosphorothioate) (Figure 1) has been widely used for pest control since its synthesis in 1959.^1,2^ Usage of FNT (also marketed as Metation, Folithion, and Sumithion), though prohibited in some countries or areas (i.e., European Union), is still (restrictively) permitted in some parts of the world.^3^ Due to agricultural activities and urbanization, a worldwide FNT residue has been reported in soils, waters, and crops,^4−6^ posing health risks for general populations through food products and drinking water.^1,7^

Following oral exposure in mammals, FNT is rapidly and almost completely absorbed and well distributed over the body especially to the liver and blood.^1^ FNT is predominately cleared by hepatic cytochromes P450 (CYP450s).^1,2^ As shown in Figure 1, CYP450s are capable of detoxifying FNT to 3-methyl-4-nitrophenol (MNP) and dimethylthiophosphate (DMTP), but they also bioactivate FNT to its oxon analogue fenitrooxon (FNO), which is a more potent inhibitor toward acetylcholinesterase (AChE) than FNT.^8,9^ A further paraoxonase 1 (PON1)-catalyzed detoxification (hydrolysis) of FNO to MNP and dimethylphosphate (DMP) takes place in both liver and blood. Subsequently, the produced MNP, DMTP, and DMP from FNT and FNO are excreted from the body via urine.^1,2^

Acute exposure to FNT will cause neurotoxic symptoms in mammals due to AChE inhibition and subsequent acetylcholine accumulation at synaptic clefts.^10^ Several no-observed-adverse-effect levels (NOAELs) derived from different *in vivo* rat or human toxicity data sets have been reported by different organizations, even though the same endpoint (erythrocyte AChE inhibition) was chosen. Specifically, NOAELs of 0.25 and 1.3 mg/kg BW for rats have been set by the US Environmental Protection Agency (US EPA)^11^ and European Food Safety Authority (EFSA),^1^ respectively, and NOAELs of 0.33 and 0.36 mg/kg BW for humans have been derived by Australian Pesticides and Veterinary Medicines Authority (APVMA)^12^ and the Joint Food and Agriculture Organization of the United Nations/World Health Organization Meeting on Pesticide Residues (JMPR),^13^ respectively. By further applying uncertainty factors accounting for the interspecies and/or interindividual differences to these NOAELs, acute reference doses (ARfDs) have been estimated, ranging from 0.0025 mg/kg BW/day (US EPA)^11^ to 0.04 mg/kg BW/day (JMPR).^13^ Due to the large variations among these ARfDs, additional studies are necessary to derive a point of departure (POD) to support the risk assessment for acute FNT exposure.

New approach methodologies are currently explored in accordance with the principles of Next Generation Risk Assessment for chemical exposures to reduce the reliance on animal approaches.^14,15^ As an example, physiologically based kinetic (PBK) modeling-facilitated quantitative *in vitro* to *in vivo* extrapolation (QIVIVE) has been widely employed for the prediction of the *in vivo* toxicity of chemicals with various endpoints,^16,17^ including AChE inhibition following acute exposure to organophosphate pesticides in both rats and humans.^18−21^ PBK models mathematically describe the body tissues as interconnected compartments linked by the blood, and can simulate time profiles of chemical concentrations in tissues under certain dose levels.^16,22,23^ Using the PBK modeling-based QIVIVE approach, *in vitro* concentration-based toxic effects can be converted to corresponding *in vivo* dose-dependent responses, supporting the derivation of PODs for risk assessment without generating new animal data.^16−21^

To predict acute toxicity following oral FNT administration in both rats and humans, the PBK modeling-facilitated QIVIVE approach was employed in the present study. The rat and human PBK models for FNT and its major metabolites were developed by integrating available physiological parameters, and chemical-specific data that were obtained *in silico* and *in vitro*. Inhibition of erythrocyte AChE in rat and human blood was determined *in vitro*. Then, the corresponding *in vivo* dose–response relationships, obtained by converting *in vitro* AChE inhibition data via QIVIVE, were used to perform a benchmark dose (BMD) analysis to derive PODs for rats and humans. By comparing the obtained PODs with available NOAELs,^1,11−13^ the performance of this approach was evaluated, and insights into the potential interspecies susceptibility toward acute FNT toxicity were provided.

## 2 Materials and Methods

### 2.1 *In Vitro* Incubations for Deriving Kinetic Parameters

*In vitro* incubations were performed to obtain kinetic parameters for the CYP450s-catalyzed conversion of FNT to FNO and MNP using gender-mixed rat and human liver microsomes. For the PON1-dominated detoxification of FNO to MNP, *in vitro* incubations were conducted with gender-mixed liver microsomes or plasma from rats and humans. Sample extraction with diisopropyl ether was performed before UPLC-PDA analysis. More details on *in vitro* incubations, sample extraction, UPLC-PDA analysis, and calculation of kinetic parameters are provided in the Supporting Information.

### 2.2 *In Vitro* AChE Inhibition Assay

Inhibition of rat and human erythrocyte AChE by FNT and FNO was determined using rat and human blood. Additionally, *in vitro* AChE inhibition assay was also performed by exposing recombinant human AChE and self-prepared rat erythrocyte AChE to FNT and FNO in a low-protein medium to investigate the potential effect induced by blood matrix. Details are provided in the Supporting Information.

### 2.3 PBK Model Establishment

**Figure 2:**
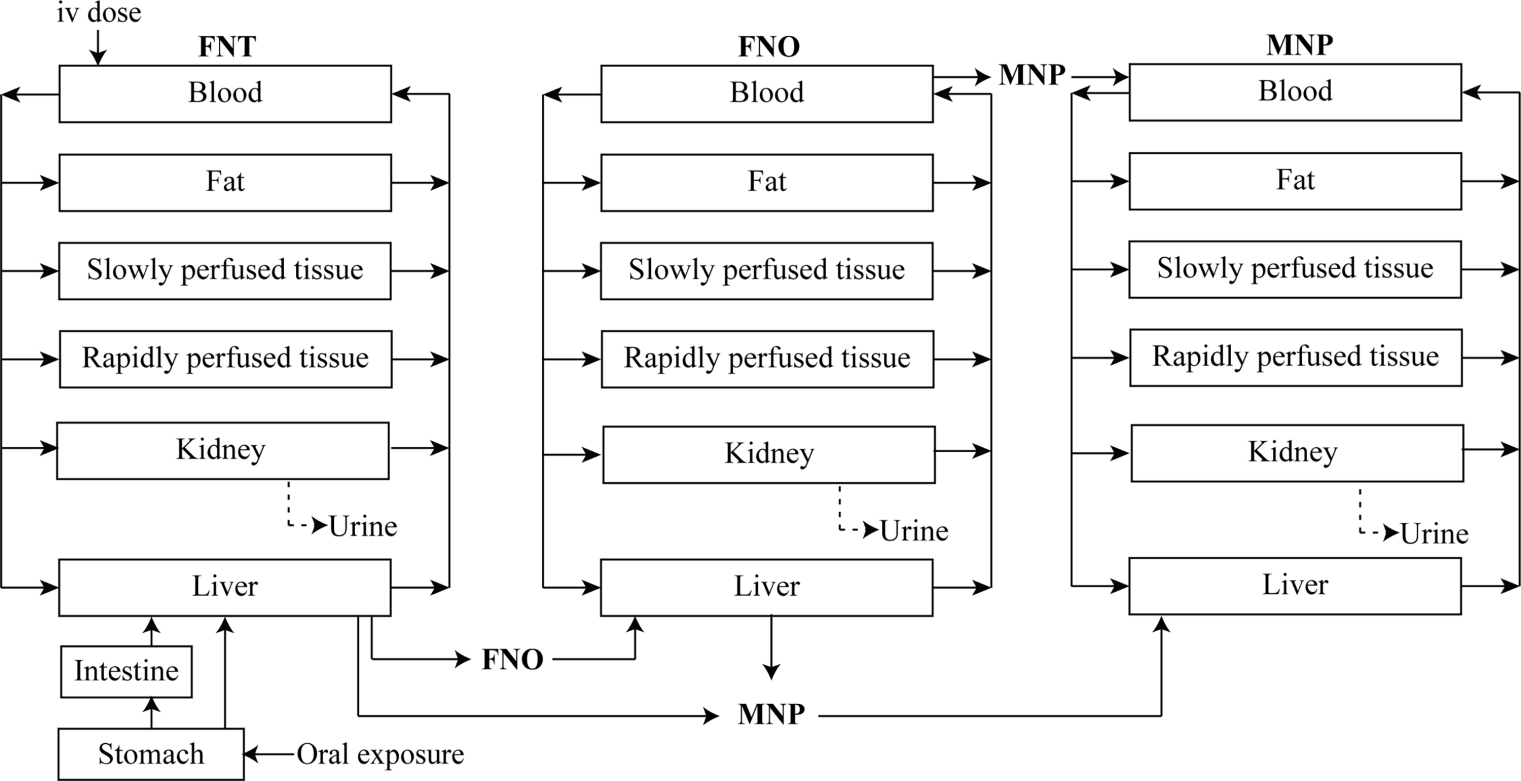
Schematic diagram of the PBK model for FNT with submodels for its major metabolites FNO and MNP.

The PBK model for diazinon^20^ was used as a starting point for establishing PBK models describing the toxicokinetics of FNT and its major metabolites (FNO and MNP) in rats and humans. Side-products DMTP and DMP formed during the conversion of FNT and of FNO to MNP, respectively, are not explicitly included in the current models, as they are not toxicologically relevant,^24^ and their exclusion will not affect the model performance. Adaptations have been made to make the model more physiologically plausible by adding a submodel to describe tissue distributions of MNP, and by using glomerular filtration rate instead of fitted excretion rate constant for urinary elimination. As presented in Figure 2, the model contains two submodels for metabolites, and each (sub)model includes liver, kidney, blood, fat, slowly perfused tissue, and rapidly perfused tissue compartments. Species-specific physiological parameters are summarized in Table S1. An oral exposure route was included in the model considering that general populations are predominantly exposed to FNT through food products and drinking water. An intravenous (iv) route was also added into the model to enable the evaluation of model performance by comparison to available kinetic data sets in rats upon iv dosing.^25^

The fraction of the dose absorbed (Fa) following oral exposure to FNT was set at 0.9 for rats^1^ and 0.7 for humans.^2^ A two-compartment gastrointestinal tract model consisting of a stomach and an intestinal compartment was used to describe oral absorption. Since absorption rate constants for FNT were not available, the relevant constants for diazinon were used considering that these two organophosphate pesticides have similar lipophilicity^26^ and are both passively absorbed. The rate constants describing the absorption from stomach to liver (*k*_a_S), absorption from intestine to liver (*k*_a_I), and transfer from stomach to intestine (*k*_s_I) were as follows: a *k*_a_S of 0.1/h for rats and 0.32/h for humans, along with a *k*_a_I of 0.59/h and a *k*_s_I of 0.48/h for both rats and humans.^20^

Distribution of FNT, FNO, and MNP across tissues was described by tissue:blood partition coefficients (*P*). The lipophilicity (log *P*) and acid–base properties (p*K*_a_) of these chemicals were precited using an online platform MolGpKa,^27^ as relevant experimental data are limited (Table S2). Then, these predicted values were used as inputs for the online QIVIVE tool (version 2.0)^28^ for predicting the fraction unbound in plasma, and tissue:plasma partition coefficients. Partition coefficients of FNT and FNO were predicted with the Berezhkovskiy method.^29^ The Rodgers and Rowland method^30^ was chosen for MNP, given that it is ionized in physiological conditions and this method takes into account the impact of drug ionization on partitioning.^31^ The blood plasma ratio (BPr) was assumed to be 0.55 for neutral (FNT and FNO) and acidic compounds (MNP), as suggested in the literature^32^ when experimental data are unavailable. Thus, corresponding tissue:blood partition coefficients for FNT, FNO, and MNP were obtained by dividing tissue:plasma partition coefficients by the BPr to correct for the difference in compound distribution between blood and plasma (Table S1).

The CYP450s-catalyzed biotransformation of FNT was assumed to occur only in the liver where these enzymes are predominantly expressed,^33^ and the resulting FNO and MNP were transferred to the corresponding submodels. In the FNO submodel, the detoxification of FNO to MNP by PON1 in both the liver and blood compartments was incorporated, and the formed metabolite MNP was transferred to the MNP submodel. Given that only the unbound fraction of chemicals is assumed to be available for the biotransformation,^34^ unbound concentrations of FNT and FNO in liver and blood were derived with unbound fraction factors as illustrated in the model code (Supporting Information). Kinetic parameters related to bioactivation and detoxification were obtained from *in vitro* incubations with liver microsomes or plasma. The *K*_m_ determined *in vitro* was postulated to be equal to the *K*_m_*in vivo*. *In vitroV*_max_ values were extrapolated to the corresponding *in vivo* values in liver, using liver microsomal protein yield scaling factors of 35 and 32 mg microsomal protein/g liver for rats and humans, respectively.^35,36^ Plasma *in vivoV*_max_ values were extrapolated from *in vitroV*_max_ values with determined plasma protein concentrations (Supporting Information) for rats (59 mg of protein/mL) and humans (66 mg protein/mL). Intestinal metabolism of FNT was not included in our model, given that its contribution comparing to the total hepatic metabolism is probably negligible as reported for chlorpyrifos and diazinon.^20,37^

Urinary elimination of FNT, FNO, and MNP from the systemic circulation was described as the glomerular filtration rate times the venous blood concentration leaving the kidney compartment. The glomerular filtration rates in rats and humans used in the models were 5.2 and 1.8 mL/min/kg BW, respectively.^38^

The model was coded and employed with Berkeley Madonna (version 10.2.8, UC Berkeley, CA, USA), applying Rosenbrock’s algorithms for stiff systems (Supporting Information).

### 2.4 PBK Model Evaluation

To evaluate the performance of the established rat and human PBK models, comparisons were made between model predictions and reported *in vivo* rat and human kinetic data (Table S3), including time profiles of blood FNT concentrations and cumulative urinary MNP excretion amounts upon oral or iv administrations. TechDig 2.0 was used to extract and convert graphic data from the reported *in vivo* studies to a numerical format.

A local sensitivity analysis was performed to identify the influential parameters on the maximum blood FNO concentration (Supporting Information), as FNO is a more potent AChE inhibitor compared to its precursor FNT, and its internal concentration is relevant for the toxicity prediction following acute FNT exposure (see section 3.3).

### 2.5 Model Application: QIVIVE

For toxicity prediction, the maximum blood FNO concentration was used as a dose metric relating AChE inhibition to acute FNT exposure (see section 3.3). Since the *in vitro* AChE inhibition under increasing FNO concentrations was tested in rat and human blood, these nominal concentrations could be used directly as the biological effective concentrations (*in vivo* maximum blood FNO concentrations), and no extra correction for differences in *in vitro* and *in vivo* protein binding was deemed necessary. Then the corresponding FNT dose levels resulting in these *in vivo* concentrations were derived via PBK modeling-facilitated reverse dosimetry. In doing so, the *in vitro* concentration–response relationship was converted to the predicted *in vivo* dose-dependent erythrocyte AChE inhibition, which was then compared to available *in vivo* toxicity data in rats and humans.^8,39^

### 2.6 BMD Analysis

BMD modeling was used to derive POD values from the predicted *in vivo* dose-dependent data sets for rats and humans. A BMD value resulting in a 10% benchmark response (BMR) change with lower 95% confidence limit was defined as BMDL_10_, and the obtained values for rats and humans were evaluated against the reported NOAELs.^1,11−13^ Details and results of BMD analysis are provided in the Supporting Information.

## 3 Results

### 3.1 *In Vitro* Kinetic Parameters

**Figure 3:**
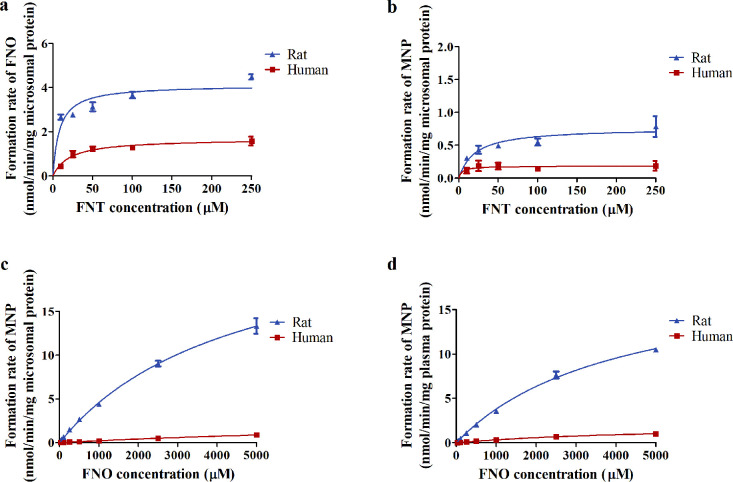
*In vitro* CYP450s-mediated formation of (a) FNO and (b) MNP in incubations with gender-mixed rat or human liver microsomes upon increasing FNT concentrations. *In vitro* PON1-mediated formation of MNP in incubations with (c) gender-mixed rat or human liver microsomes and (d) gender-mixed rat or human plasma upon increasing FNO concentrations. Results are presented as means ± SEM from three independent experiments. The derived *in vitro* kinetic parameters (*V*_max_, *K*_m_, and catalytic efficiency) for rats and humans were summarized in Table S4. Due to solubility limits, the highest FNO concentration tested was 5000 μM. Although curves in (c) and (d) did not reach plateaus, the correlation coefficient (*r*^2^ > 0.97) for the fit of the Michaelis–Menten equation and the experimental data indicated an adequate fit of *K*_m_ and *V*_max_ values.

The biotransformation of FNT and FNO was determined from *in vitro* incubations with gender-mixed rat and human liver microsomes or plasma. As shown in Figure 3, for rats and humans, FNT bioactivation was faster than its detoxification and PON1-mediated FNO hydrolysis was comparable in liver and plasma. Interspecies differences in toxicokinetics were notable. The unscaled catalytic efficiency of the CYP450s-catalyzed bioactivation was 6.4-fold higher in rats than in humans, though their detoxification efficiencies were comparable (Table S4). For the detoxification of FNO by liver and plasma PON1, a faster hydrolysis was observed in rats than in humans (Figure 3).

### 3.2 PBK Model Evaluation

The established PBK models were first evaluated by comparing model predictions with available *in vivo* kinetic data (Table S3) to ensure their adequate performance. According to the guideline^40^ from the World Health Organization, model predictions that are within 2-fold difference as compared to the experimental data are considered to be well-fitted, while a larger difference is also acceptable especially when experimental data sets obtained from different studies show inconsistencies.

**Figure 4:**
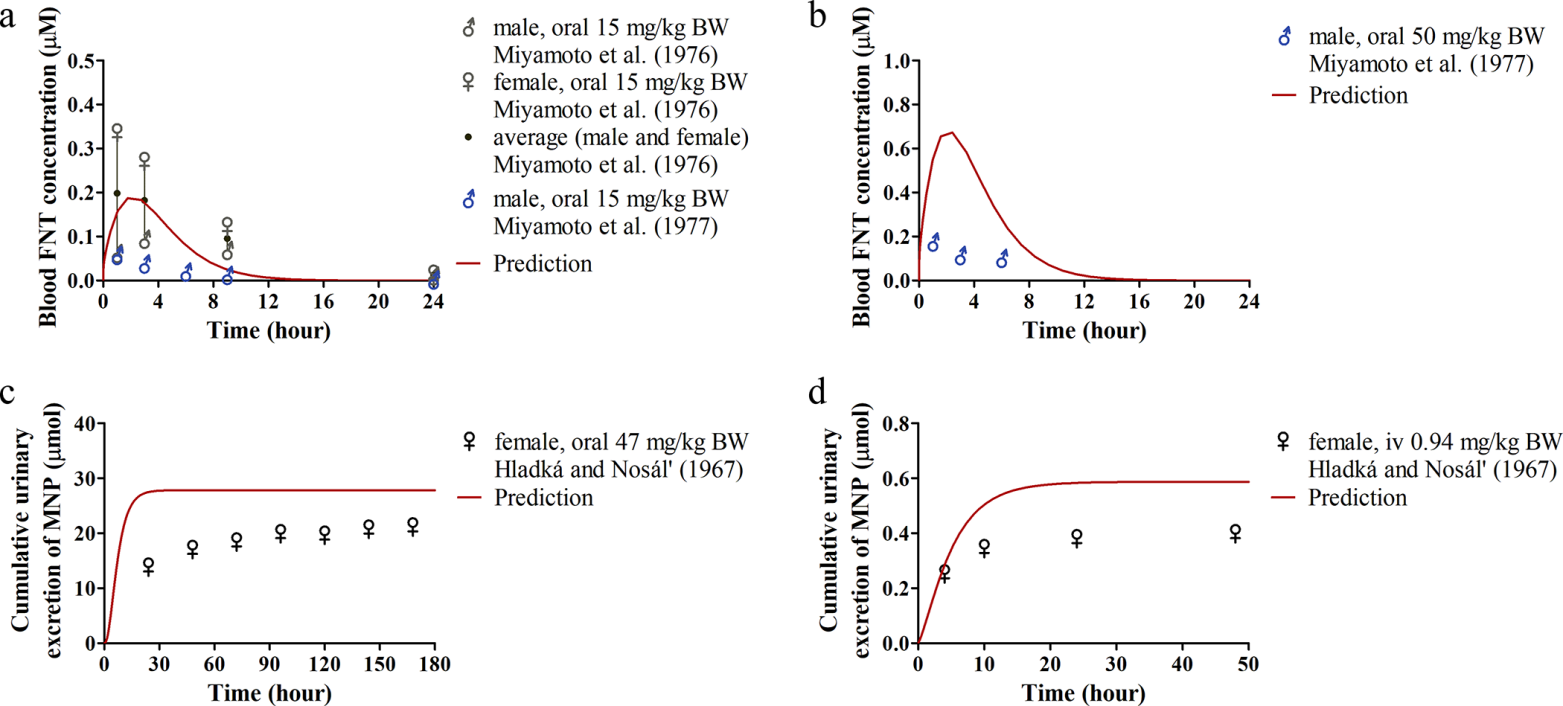
Comparisons between reported *in vivo* rat data and predictions made from the gender-mixed rat PBK model. Predicted and reported (a) time-dependent blood FNT concentrations in male and female rats upon single oral administration of FNT at 15 mg/kg BW;^41,42^ (b) time-dependent blood FNT concentrations in male rats upon single oral administration of FNT at 50 mg/kg BW;^42^ (c) time-dependent cumulative urinary MNP excretion in female rats upon single oral administration of FNT at 47 mg/kg BW;^25^ and (d) time-dependent cumulative urinary MNP excretion in female rats upon iv administration of FNT at 0.94 mg/kg BW.^25^

For the rat model, time-based rat blood FNT concentrations following oral FNT administration were available.^41,42^ Interestingly, potential gender-related differences in rats were noticed,^41^ where at the same dose level and time point, blood FNT concentrations in female rats were higher than those in males, and also reached the peak concentration within a shorter period (Figure 4a). In addition, variations between *in vivo* studies^41,42^ were observed, where the disappearance of FNT in blood differed, though in both studies the male rats were exposed to the same dose (Figure 4a). Our predicted blood FNT concentrations from the gender-mixed model fit well with the averaged data of male and female rats (Figure 4a). The model overpredicted the concentrations collected solely from males (Figure 4a,b), probably due to gender-related differences. In another *in vivo* study,^25^ cumulative urinary MNP excretion was monitored in female rats after oral or iv administration of FNT. Our predicted MNP excretion matched the respective experimental data well, and the differences were within 2.0 and 1.5-fold, respectively (Figure 4c,d).

**Figure 5:**
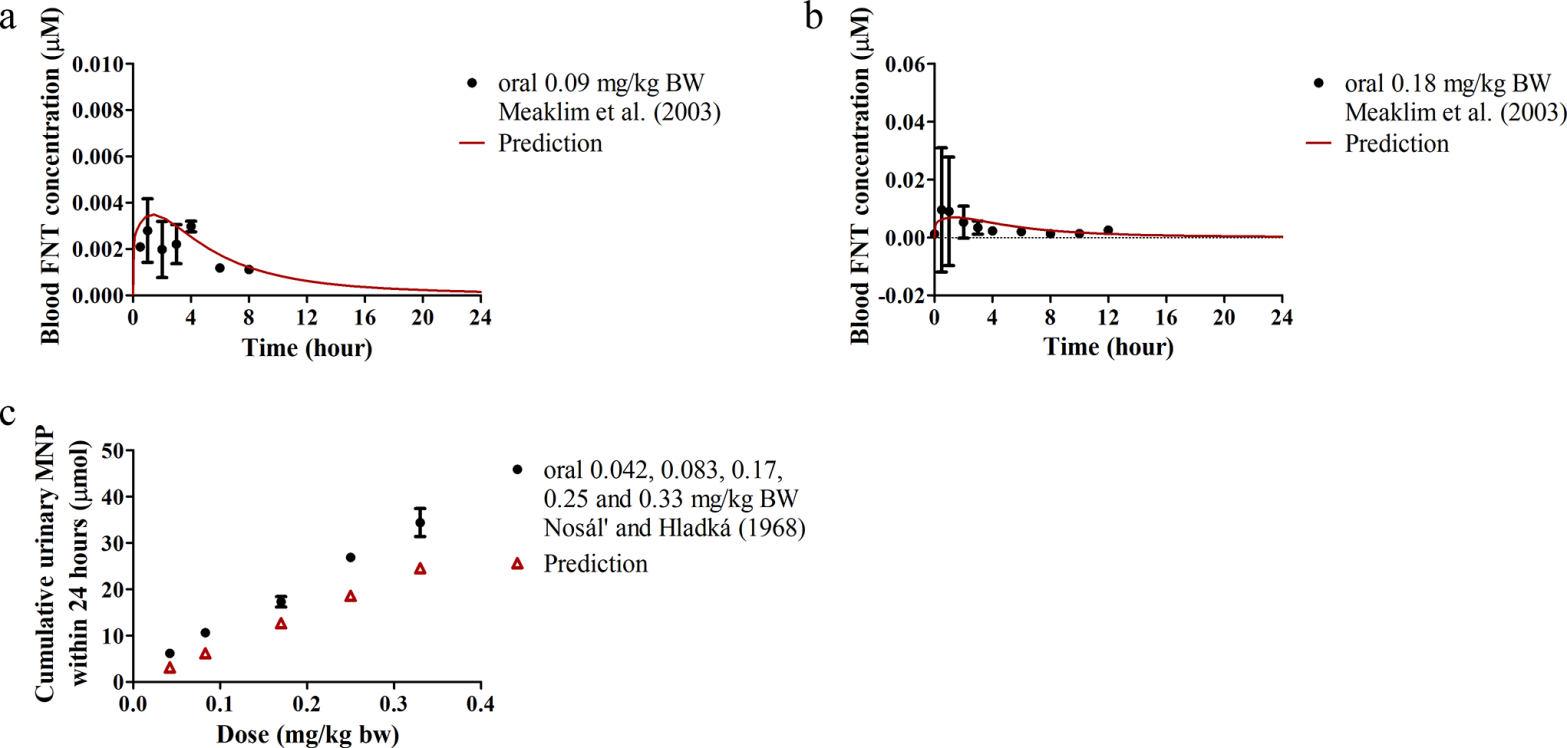
Comparisons between human PBK model predictions and reported *in vivo* human data. Predicted and reported (a) time-dependent blood FNT concentrations in humans upon single oral administration of FNT at 0.09 mg/kg BW;^43^ (b) time-dependent blood FNT concentrations in humans upon single oral administration of FNT at 0.18 mg/kg BW;^43^ and (c) dose-dependent cumulative urinary MNP excretion within 24 h in humans upon single oral administration of FNT at 0.042, 0.083, 0.17, 0.25, and 0.33 mg/kg BW.^39^ Data collected from the literature are presented as means ± SEM (where available).

For the human model, time profiles of blood FNT concentrations and urinary MNP amounts following a single oral administration of FNT were available. In a volunteer study,^43^ 12 participants (8 males and 4 females) received FNT formulated as a capsule together with food for 4 days, with the daily dose being administrated as two divided doses with a 12 h interval. The time-based blood FNT concentrations within the first 12 h following a single FNT exposure were collected, and large interindividual variations were noted (Figure 5a,b). The differences between the averaged *in vivo* data and model predictions were within 1.7 and 2.2-fold at dose levels of 0.09 and 0.18 mg/kg BW, respectively (Figure 5a,b). In another volunteer study,^39^ FNT was prepared with olive oil in gelatin capsules and was given to 24 individuals (gender unspecified), and the administrated doses (0.042, 0.083, 0.17, 0.25, and 0.33 mg/kg BW) were calculated with an average body weight of 60 kg as used by JMPR^2^ and APVMA.^12^ The cumulative urinary MNP excretion within 24 h following these five dose levels was reported, and the differences between the reported average values and our predictions were within 2.0-fold (Figure 5c).

Taking all comparisons into consideration, we concluded that the rat and human PBK models were able to provide adequate predictions of time-dependent blood FNT concentrations and urinary MNP amounts. Following a local sensitivity analysis to have an overview of the influential parameters on the model output (Figure S1), the rat and human PBK models were subsequently used for QIVIVE.

### 3.3 Prediction and Evaluation of *In Vivo* Dose-Dependent AChE Inhibition

**Figure 6:**
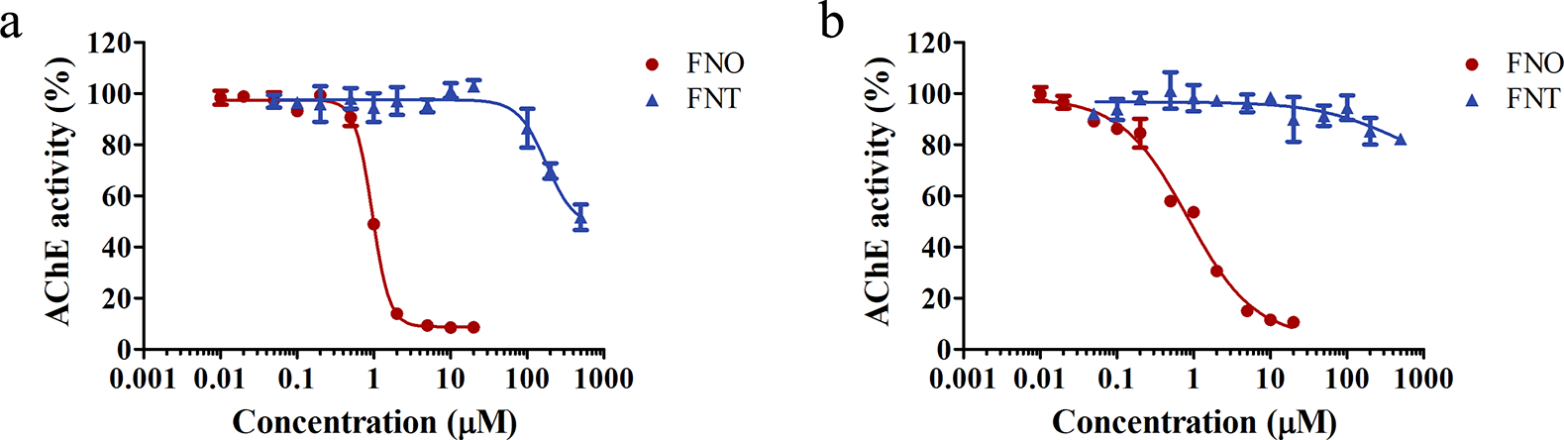
Blood AChE activity upon incubation with increasing concentrations of FNT and FNO in (a) rats and (b) humans. Results are presented as means ± SEM from three independent experiments. The background AChE activities in rat and human blood without exposure to compounds and solvent were 0.5 and 4.1 U/mL, respectively.

The *in vitro* concentration-dependent AChE inhibition in blood under increasing concentrations of FNT and FNO was determined for rats and humans. As shown in Figure 6, FNT is corroborated to be a weak AChE inhibitor, with a concentration resulting in 50% inhibition (IC_50_) of about 500 μM for rats and an IC_50_ greater than the highest tested concentration (500 μM) for humans. FNO is a more potent inhibitor as compared with its precursor FNT, and comparable IC_50_ values of FNO were obtained for rats (0.95 μM) and humans (0.84 μM). Given that FNO could induce inhibition to rat and human erythrocyte AChE at a much lower concentration than FNT (Figure 6), and that its blood concentration is predicted to be higher than that of FNT in both rats and humans at an FNT dose range relevant for risk assessment (Figure S2), it can be concluded that the contribution of FNT to AChE inhibition is negligible. Therefore, the maximum FNO concentration in blood is relevant for the acute toxicity following FNT exposure and was used for subsequent QIVIVE.

**Figure 7:**
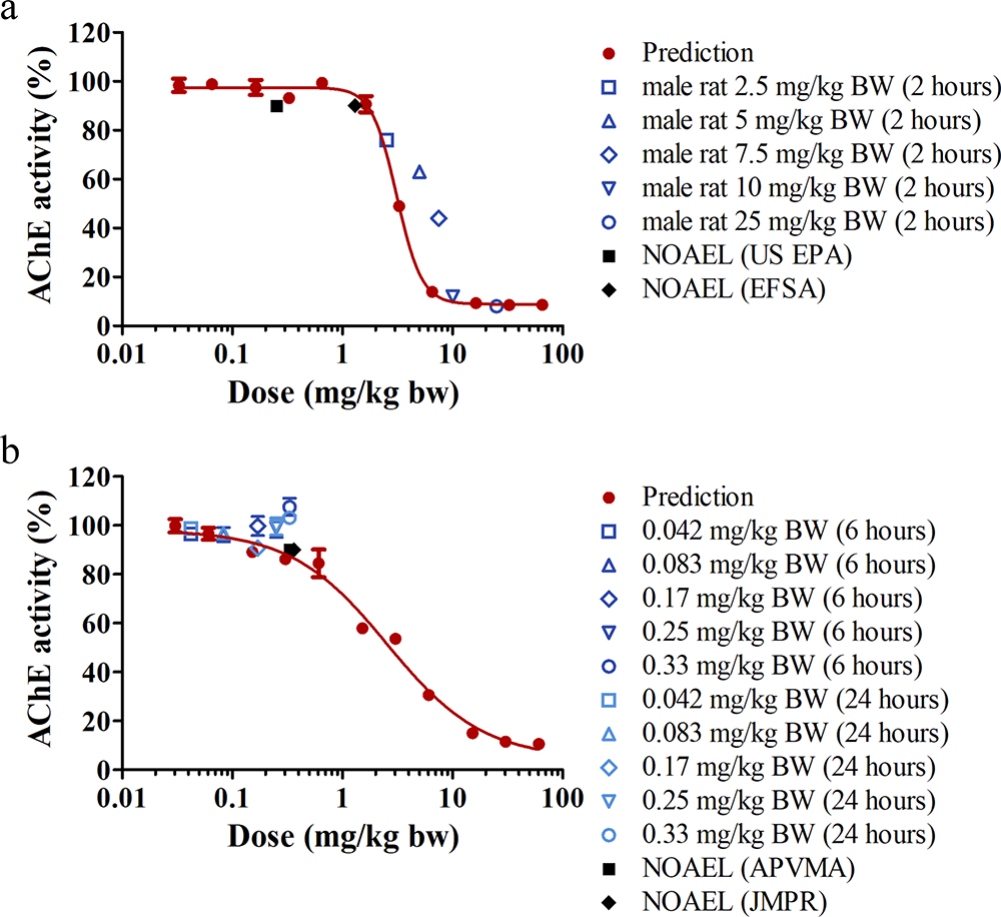
Predicted *in vivo* dose-dependent AChE activity by PBK modeling-facilitated QIVIVE for (a) rats and (b) humans upon a single oral administration of FNT. Available *in vivo* erythrocyte AChE inhibition data in male rats^8^ and humans^39^ are presented as means ± SEM (where available). Reported NOAELs^1,11−13^ for rats and humans are plotted as black square or diamond by assuming that these values represented the dose levels that resulted in 10% inhibition in AChE activity.

The *in vitro* FNO concentration-based AChE inhibition obtained using rat and human blood were converted to corresponding *in vivo* FNT dose-dependent response curves via PBK modeling-facilitated QIVIVE and then compared to available *in vivo* AChE inhibition data (Figure 7). For rats, the erythrocyte AChE activity was measured 2 h after oral administration of FNT at dose levels of 2.5, 5, 7.5, 10, and 25 mg/kg BW in males,^8^ and its activity decreased rapidly with increasing FNT dose levels (Figure 7a). Our predictions are comparable with the *in vivo* data and describe the fast dropping of AChE activity well (Figure 7a). One human study^39^ reporting AChE inhibition following a single FNT administration was found, where relatively low dose levels (0.042, 0.083, 0.17, 0.25, and 0.33 mg/kg BW) were orally administered to 24 participants (gender unspecified), and the erythrocyte AChE activity was monitored at 6 and 24 h after exposure. The predicted curve for humans confirms that at the dose levels reported in the study the AChE inhibition is indeed limited (Figure 7b).

### 3.4 POD Derivation and Evaluation

By performing BMD analysis on the predicted rat and human *in vivo* dose-dependent erythrocyte AChE inhibition curves, a rat and a human BMDL_10_ were derived as PODs. The results (Table 1) indicate that the predicted POD for humans is 5-fold lower than that of rats, suggesting that humans are more susceptible than rats toward the acute toxicity induced by FNT.

**Table 1:** Comparisons of Reported NOAEL and Predicted BMDL_10_ Values

**POD (mg/kg BW)**	**reported NOAEL**		**predicted BMDL_10_**	
	rat	human	rat	human
**FNT**	0.25 (US EPA); 1.30 (EFSA)	0.33 (APVMA); 0.36 (JMPR)	1.30	0.26

Different NOAELs used by several organizations for setting ARfDs are summarized in Table 1. A NOAEL of 1.3 mg/kg BW was reported by EFSA^1^ from a 90 day rat study^44^ considering the impaired body weight gain and reduction in erythrocyte and brain cholinesterase activity as the critical effects. The US EPA^11^ reported a 5.2-fold lower NOAEL (0.25 mg/kg BW) based on the measured erythrocyte AChE inhibition after 2 weeks of FNT dosing in a 92 week rat study.^45^ Different from EFSA and the US EPA, APVMA and JMPR derived NOAELs based on available human data. APVMA^12^ established a NOAEL of 0.33 mg/kg BW from a single dose human study,^39^ based on the absence of any inhibition of plasma and erythrocyte cholinesterase activity at this highest dose level tested in human volunteers. JMPR^13^ identified a 4 day study in human volunteers^43^ as the most suitable study, and set a NOAEL of 0.36 mg/kg BW where no subject showed decreases in erythrocyte AChE activity at this highest dose level tested. Our predicted POD for rats is comparable to the NOAEL derived by EFSA, and the predicted POD for humans is in line with the current human NOAELs (Table 1).

## 4 Discussion

This work aimed to use an approach in accordance with the 3R principles (Replacement, Reduction, and Refinement) of animal testing to predict dose levels at which humans would not be at risk for acute neurotoxicity due to erythrocyte AChE inhibition induced by an oral exposure to FNT. To this end, PBK models for both rats and humans were first established and validated. Subsequently, the models were used for QIVIVE to convert the *in vitro* determined concentration-dependent AChE inhibition in rat and human blood to *in vivo* dose-dependent curves, from which rat and human BMDL_10_ values were derived as PODs, which were comparable with the current rat and human NOAELs.

Following exposure to organophosphate pesticides, liver CYP450s-mediated bioactivation and detoxification take place.^46^ For FNT, the bioactivation instead of its detoxification is found to be more efficient in rats and humans (Figure 3), as opposed to the results found for chlorpyrifos and diazinon.^18,20^ Several CYP450s including 1A2, 2B6, 2C19, and 3A4 were reported to participate in both bioactivation and detoxification of organophosphate pesticides,^19,20^ while specific information on the CYP450s involved in FNT metabolism is limited. Levi et al.^47^ purified two chemically induced isoforms (named as P-450 PB and P-450 BNF) from livers of mice that were pretreated orally or intraperitoneally with phenobarbital (PB) or β-naphthoflavone (BNF), inducers for CYP2B9/10 and 1A1/2 in mice, respectively.^48,49^ In their study, P-450 PB and P-450 BNF were reported to produce higher amounts of FNO compared to MNP production. Given that CYP1A and 2B genes presumably carry out similar or identical functions in both humans and mice,^50^ CYP1A2 and 2B6 in humans are speculated to mainly participate in the bioactivation of FNT. This assumption is in line with the observations from a study,^51^ where methyl parathion, a structural analog of FNT (methyl parathion lacks a methyl group on the aryl group) was incubated with several recombinant human CYP450s, and 1A2 and 2B6 were found to mainly participate in its bioactivation. Additionally, CYP2D6 might also participate in the FNT biotransformation, given that the classical substrates for CYP2D6 are aryl- or alkyl-amines with ionized nitrogen at physiological conditions.^52^ Due to the varied expression and activity of the CYP450s^33^ that might participate in the bioactivation and detoxification of FNT, toxicokinetic differences among individuals could be expected, which might be an explanation to the interindividual variances reported in the blood FNT concentrations under the same exposure level.^43^

PON1 is primarily synthesized in the liver and secreted into the blood,^53^ it is active as a main esterase in hydrolyzing oxon analogues rather than the thiophosphate precursors like FNT in this study.^46^ Hydrolysis efficiency of PON1 toward oxons varies among organophosphate pesticides, as a slight change in their leaving groups can affect the orientation of the oxon in the active site and hence their conversion.^54^ For example, the PON1-catalyzed detoxification of methyl paraoxon was found to be less efficient than that of CPO in both rats and humans.^54,55^ This observation is in agreement with the lower PON1 catalytic efficiency toward FNO (Table S4) as compared to that of CPO,^19^ considering that methyl paraoxon and FNO are highly similar in structure. Similar to CYP450s, the activity of PON1 shows interindividual differences,^56^ while relevant information is limited on how it will affect the resulting toxicity among individuals following acute FNT exposure.

In addition to liver and plasma clearance, renal clearance of FNT, FNO, and MNP was also included in the established PBK models. Currently, glomerular filtration is considered as the major elimination route. MNP can be cleared renally by glomerular filtration;^57^ however, the use of glomerular filtration for FNT and FNO might overestimate their renal clearance to some extent, as passive reabsorption might occur for chemicals with high lipophilicity.^57^ This might be an explanation for the underestimation of urinary MNP excretion in humans (Figure 5c), as the formation of MNP from FNT and FNO might be underestimated due to the probable overestimation of the elimination of FNT and FNO in urine. Further studies are necessary to enable refinement of the modeling to renal elimination of organophosphate pesticides.

AChE is encoded by a single gene.^58^ In addition to neuromuscular junctions, AChE is also present in the blood (erythrocyte AChE), though its physiological function to the erythrocytes is still unclear.^59^ The mechanism of acute neurotoxicity following organophosphate pesticide exposures is the inhibition of neuronal AChE; however, relevant *in vivo* data for it are usually unavailable especially for humans. In the absence of this information, the inhibition of erythrocyte AChE is considered to be an acceptable surrogate endpoint in scientific studies^18−21^ and by regulatory bodies^60^ because erythrocyte AChE is more sensitive than the neuronal AChE,^61^ suggesting that a conservative POD could be derived with this endpoint.

FNO shows a similar inhibition potency toward erythrocyte AChE in rats and humans, while the contribution of FNT to AChE inhibition is negligible (Figure 6). Interestingly, the activity of self-prepared rat erythrocyte AChE and recombinant human AChE is notably inhibited by both FNT and FNO (Figure S3), and the obtained IC_50_ values for FNO are 5.3 and 3.4-fold lower than those obtained in rat and human blood, respectively. Similar observations are also noted for diazinon and its oxon, where diazinon inhibited AChE activity when measuring with recombinant human AChE in a low-protein medium,^20^ but exerted a limited influence on AChE inhibition when measuring with human blood.^62^ As for diazinon oxon, a lower IC_50_ was derived with the recombinant enzyme^20^ than that obtained with blood.^62^ These observations indicate that specific factor(s) (i.e., plasma proteins) present in the blood might affect the interaction between AChE and organophosphate pesticides. The AChE inhibition data obtained from rat and human blood were used for QIVIVE in the present study, as testing with blood could simulate the physiological conditions better, and also takes into account the potential differences induced by species-dependent blood matrix effects (Figure 6).

Species-relevant toxicokinetic differences were observed in the current study, where rats appeared to be more efficient in FNT and FNO conversion than humans (Figure 3). Interestingly, when exposed to the same dose levels, comparable maximum blood FNO concentrations were predicted for the two species (Figure S2), indicating that there are other reasons that cause different POD predictions for rats and humans. Though similar IC_50_ values for FNO toward rat and human erythrocyte AChE activity are derived, the rat and human *in vitro* AChE inhibition curves especially in the low-concentration region are different. Specifically, in the *in vitro* assay, the AChE activity in rat blood is initially resistant to increasing FNO concentrations before a fast dropping, while the human AChE activity decreases progressively in the whole FNO concentration range (Figure 6). After conversion to the *in vivo* dose–response curve (Figure 7) for BMD analysis, the low-dose region becomes particular important as this is the region for POD (BMDL_10_) derivation.^63^ Plasma carboxylesterase might be a factor related to the low-dose differences observed between rat and human blood, as this enzyme could protect AChE by scavenging oxon analogues of organophosphate pesticides,^64^ while human plasma contains no carboxylesterase as opposed to the rat plasma.^65^

Additionally, gender-related differences were noticed between male and female rats. Compared to males, female rats have a slower FNT biotransformation at the same exposure level.^41^ However, further information on the resulting maximum blood FNO concentration is limited. For humans, relevant information about gender-dependent FNT depletion and FNO formation is unavailable. More studies are necessary to clarify the relationship, if it exists, between gender-related expression of specific enzymes and the resulting toxicity.

Still, further work could be made to improve the current approach by taking into consideration the following limitations. First, only FNO and MNP as major metabolites of FNT were included in the biotransformation (Figure 1), while other metabolites might also be formed. For example, demethyl-FNT and demethyl-FNO were detected in the urine during 48 h after an oral administration of FNT (15 mg/kg BW) in rats, mice, rabbits, and dogs, and their relative amounts were species-specific.^41^ No relevant information for humans is available. Interestingly, demethyl-FNT and demethyl-FNO were not found in our *in vitro* incubations with rat or human liver microsomes, indicating that their formation, at least *in vitro*, is inefficient compared to the formation of FNO and MNP. Second, the MNP submodel included in our current PBK models only describes its tissue distribution and urinary excretion, without capturing its further glucuronidation and sulfation in the liver.^41,42^ This is because the data sets used for model validation (Figures 4c,d and 5c) provided the total amount of MNP cumulated in the urine, of which the conjugated MNP was released by acidic hydrolysis during sample preparation.^66,67^ Third, ethopropazine was used as an inhibitor to butyrylcholinesterase in the *in vitro* AChE inhibition assay^62^ and this might result in a conservative POD prediction, as butyrylcholinesterase could protect erythrocyte AChE by binding with oxon analogues of organophosphate pesticides.^68^ The potential protection from butyrylcholinesterase especially for humans, who have a much higher plasma butyrylcholinesterase activity than rats,^69,70^ was not taken into account, and this might explain the gap between the predicted and reported PODs for humans (Table 1). Fourth, a common challenge exists when extrapolating from an *in vitro* endpoint to an *in vivo* apical endpoint that usually occurs on a different time scale, due to, i.e., time for toxicokinetics *in vivo*.^71^ In the future, the current model could be refined by combining it with a dynamic (D) submodel with parameters describing synthesis, degradation, inhibition, reactivation, and aging of AChE. Relevant PBK/D models have been developed for chlorpyrifos and diazinon.^72,73^ In this way, the time-dependent relationship between oxon concentrations in target tissues and the resulting AChE inhibition could be correlated. In spite of these limitations, the current approach could adequately predict toxicokinetics (Figure 4 and 5) and PODs (Table 1), suggesting that it is sufficient for hazard and risk assessment upon acute FNT exposure.

In summary, the results obtained in the current study provide a proof-of-principle for applying the PBK modeling facilitated-QIVIVE approach for predicting erythrocyte AChE inhibition induced by acute FNT exposure. This approach can be applied for other organophosphate pesticides by modifying the chemical-specific parameters used in the PBK model and QIVIVE. Considering that organophosphate pesticides will still be used worldwide for a long time, this approach is promising in deriving PODs for the risk and safety evaluation of organophosphate pesticides in accordance with the 3R principles, and could also facilitate the future development of a generic PBK model to predict the acute toxicity for a large number of organophosphate pesticides.

## Supporting Information Available

Materials and methods for *in vitro* incubations, sample extraction, UPLC-PDA analysis, plasma protein concentration determination, and *in vitro* AChE inhibition assay; local sensitivity analysis; summary of physiological, physicochemical and kinetic parameters; summary of available *in vivo* kinetic studies in rats and humans; summary of log *P* and p*K*_a_ values; details of BMD analysis; PBK model codes (PDF)
