# Supplementary material for: Acetylcholinesterase Inhibition in Rats and Humans Following Acute Fenitrothion Exposure Predicted by Physiologically Based Kinetic Modeling-Facilitated Quantitative In Vitro to In Vivo Extrapolation
Source: Environ Sci Technol. 2023 Nov 27;57(49):20521–31. doi: 10.1021/acs.est.3c07077 (PMC10720383; doi:10.1021/acs.est.3c07077)
Supplement: Supplementary file 1 — es3c07077_si_001.pdf [file es3c07077_si_001.pdf]

SUPPORTING INFORMATION

Acetylcholinesterase Inhibition in Rats and Humans Following Acute Fenitrothion Exposure Predicted by Physiologically Based Kinetic Modeling-Facilitated Quantitative *In Vitro* to *In Vivo* Extrapolation

Jiaqi Chen[a], Shensheng Zhao[a], Sebastiaan Wesseling[a], Nynke I. Kramer[a], Ivonne M.C.M. Rietjens[a], Hans Bouwmeester[a]*

[a] Division of Toxicology, Wageningen University and Research, Stippeneng 4, 6708 WE, Wageningen, The Netherlands

* Corresponding author: Jiaqi Chen (jiaqi.chen@wur.nl)

29 pages, 4 Tables, 3 Figures

Supplementary materials and methods

Chemical and biological materials

FNT, acetylthiocholine iodide (ATC), 5,5'-dithiobis (2-nitrobenzoic acid) (DTNB), ethopropazine, tetraisopropyl pyrophosphoramide (iso-OMPA), perchloric acid ($HClO_4$), trifluoroacetic acid (TFA), EDTA, $MgCl_2$•$6H_2O$, $CaCl_2$•$2H_2O$, NaH_2PO_4, Na_2HPO_4, Triton-X 100, Trizma® base and diisopropyl ether (DIPE) were ordered from Sigma-Aldrich (Amsterdam, The Netherlands). FNO was purchased from FUJIFILM Wako Pure Chemical Corporation (Tokyo, Japan), MNP and reduced nicotinamide adenine dinucleotide phosphate (NADPH) from Carbosynth Ltd. (Compton, UK). Acetonitrile (UPLC/MS grade), methanol (UPLC/MS grade) and ethanol (UPLC/MS grade) were ordered from Biosolve (Valkenswaard, The Netherlands). Chlorpyrifos-oxon was ordered from TRC-Canada (Toronto, Ontario, Canada). Ultrapure water was prepared using a Sartorius Arium® Pro ultrapure water system (Göttingen, Germany).

Human liver microsomes (pooled from 150 donors, mixed gender) and pooled male rat liver microsomes (Sprague-Dawley) were purchased from Corning (Amsterdam, The Netherlands). Pooled female rat liver microsomes (Sprague-Dawley) were ordered from Sigma-Aldrich (St. Louis, MO, USA). Gender-mixed rat liver microsomes used in *in vitro* incubations were pooled from the male and female ones mentioned above. Human plasma (pooled from 25 donors, mixed gender) and rat plasma (Sprague-Dawley, mixed gender) were ordered from BioIVT (West Sussex, UK), and corresponding total protein concentrations were determined using BCA protein assay (Supporting Information). Human whole peripheral blood and rat whole blood (Sprague-Dawley) for *in vitro* blood AChE inhibition assay were ordered from CTIBiotech (Lyon, France) and Innovative Research Inc. (Novi, MI, USA), respectively, both with K_2EDTA as an anticoagulant and were gender unspecified. Rat whole blood (Sprague-Dawley rat, mixed gender, with LiHep as an anticoagulant) used for preparing erythrocyte AChE was ordered from BioIVT (West Sussex, UK). Recombinant human acetylcholinesterase (rhAChE) and bovine serum albumin (BSA) were ordered from Sigma-Aldrich (St. Louis, MO, USA). The Pierce™ BCA protein assay kit was ordered from Thermo Fisher (Landsmeer, The Netherlands).

In vitro incubations for metabolic conversions of FNT

In vitro incubations were performed to obtain kinetic parameters for the CYP450-catalyzed conversion of FNT to FNO and MNP using rat and human liver microsomes as described by Zhao et al. (2021) with modifications. Preliminary experiments were performed for optimization to ascertain that reactions were linear with respect to time and microsomal protein concentration (data not shown). To the microsomal incubations with FNT, EDTA and iso-OMPA were added as inhibitors for PON1 and B-esterases, respectively, to prevent untargeted metabolism of the formed FNO in the reaction system. Briefly, the incubations with a total volume of 200 μL contained 50 mM Tris-HCl (pH 7.4), 5 mM $MgCl_2$, 1 mM EDTA, 50 μM iso-OMPA, 1 mM NADPH, and FNT at final concentrations ranging from 10 to 250 μM

(added from 100 times concentrated stock solutions in acetonitrile). Controls were carried out by replacing NADPH with Tris-HCl. After 1-min pre-incubation in a shaking water bath at 37°C, 1 µL rat or 5 µL human liver microsomes (final concentration 0.1 and 0.5 mg microsomal protein/mL for rat and human samples, respectively) was added to initiate the reaction. After a 2-min incubation, the reaction was terminated by the addition of 20 µL ice-cold $HClO_4$ (10%, *v/v*), and samples were kept on ice until further extraction. All incubations were performed in triplicate.

***In vitro* incubations for metabolic conversions of FNO**

Incubations with liver microsomes and plasma were performed to obtain kinetic parameters for PON1-mediated detoxification of FNO to MNP based on a modified method (Zhao et al. 2021). Incubation conditions that were suitable for linearity with respect to time and microsomal protein or plasma protein concentration were defined (data not shown). The final incubation mixtures with a total volume of 200 µL contained 50 mM Tris-HCl (pH 7.4), 2 mM $CaCl_2$ as PON1 activity simulator, and FNO at final concentrations ranging from 25 to 5000 µM (added from 100 times concentrated stock solutions in acetonitrile). Controls differed from test incubations by the absence of liver microsomes or plasma, which was replaced with Tris-HCl. After 1-min pre-incubation in a 37°C water bath, 2 µL rat or 10 µL human liver microsomes (final concentration 0.2 and 1 mg microsomal protein/mL for rat and human samples, respectively) was added to initiate the reaction. For the plasma incubations, 4 µL of rat plasma or 10 µL human plasma (final concentration 1.2 and 3.3 mg plasma protein/mL for rat and human samples, respectively) was used. Reactions were terminated after incubating 10 min for rat samples or 20 min for human samples by adding 20 µL ice-cold $HClO_4$ (10%, *v/v*), and samples were kept on ice until further extraction. All incubations were performed in triplicate.

Sample extraction

DIPE extraction of FNT and its metabolites (Zhao et al. 2019; Wang et al. 2022) was performed before UPLC analysis. Briefly, 1 mL DIPE was added to the ice-cold samples and mixed well, then the upper DIPE layer containing the target compounds was transferred into glass tubes. After another two repeated extractions, all collected DIPE layers were combined and evaporated to dryness under an N_2 stream. Finally, the obtained residue was redissolved in 100 µL methanol, and subsequently analyzed with UPLC-PDA.

UPLC-PDA analysis

The quantification of FNT, FNO and MNP was conducted using a Shimadzu Nexera X2 liquid chromatography system (LC-30AD, Kyoto, Japan), connected with a photodiode array detector (SPD-M30A, Shimadzu, Kyoto, Japan). Chromatographic separation was performed with a Waters Acquity UPLC BEH C18 column (50 mm × 2.1 mm, 1.7 µm) coupled with a Waters Xbridge UPLC BEH C18 pre-column (5 mm × 2.1 mm, 2.5 µm). Column temperature was maintained at 40°C and auto-sampler

temperature at 10°C during analysis. Ultrapure water (containing 0.1% TFA, *v/v*) and acetonitrile were used as mobile phases. A 22-min linear gradient with a flow rate of 0.6 mL/min ran from 10% to 100% acetonitrile over 17 min and held at 100% acetonitrile for 1 min, then returned to 0% over 0.3 min and held at 0% acetonitrile for 1 min, finally returning to the initial conditions and remaining for 2 min before the next injection. Under these conditions the retention times of FNT, FNO and MNP were 7.86 min, 4.65 min and 3.55 min, respectively. Quantification of FNT and FNO was performed by integrating peak areas at 269 nm and for MNP at 315 nm using calibration curves ($r^2 > 0.99$) prepared with commercial standards. The limit of quantification was 0.1 µM for FNT, FNO and MNP when using an injection volume of 20 µL. During the whole work, the performance of the UPLC-PDA was stable and consistent, and no influence of the presence or absence of the biological matrix (liver microsomes or plasma) on the quantification was observed.

Calculation of kinetic parameters

The kinetic parameters for the conversions of FNT to FNO and MNP in incubations with rat and human liver microsomes, and of FNO to MNP in incubations with rat and human liver microsomes or plasma were determined by fitting the data to a standard Michaelis-Menten equation (Eq S1)

$$v = \frac{V_{max} \times [S]}{K_m + [S]} \qquad \text{(Eq S1)}$$

where v represents metabolite formation rate in nmol/min/mg microsomal protein or nmol/min/mg plasma protein, [S] the substrate concentration in µM, K_m the apparent Michaelis-Menten constant in µM, and V_{max} the apparent maximum rate in nmol/min/mg microsomal protein or nmol/min/mg plasma protein. Data were analyzed in GraphPad Prism (version 5.04, San Diego, CA, USA) and each data point was presented as the mean value ± SEM.

Protein concentration determination for rat and human plasma

Total protein concentration of both rat and human plasma was determined following the manufacturer's protocol (Thermo Fisher 2020). Briefly, 25 µL plasma sample or protein standard solution was incubated with 200 µL working reagents in a 96-well plate (Greiner Bio-One, The Netherlands) at 37ºC for 30 min, after cooling it to room temperature the absorbance at 562 nm was measured. Protein concentrations of rat and human plasma were quantified using a calibration curve prepared with the protein standard in the assay kit.

***In vitro* AChE inhibition assay with rat and human blood**

Inhibition of rat and human erythrocyte AChE by FNT and FNO was determined using the protocol from Kasteel et al. (2021). The final concentrations of FNT and FNO in rat and human blood ranged from 0.05 to 500 µM and from 0.01 to 20 µM, respectively (added from 1000 times concentrated stock solutions in ethanol). Absorbance at 436 nm was measured continuously for 60 min and 10 min to detect

the remaining AChE activity in rat and human blood samples, respectively. Assays were conducted in triplicate, and the data obtained were analyzed in GraphPad Prism (version 5.04, San Diego, CA, USA) to define the concentration resulting in 50% inhibition (IC_{50}).

In vitro AChE inhibition assay with self-prepared rat erythrocyte AChE and recombinant human AChE

Based on the method described in Zhao et al. (2021), rat erythrocyte AChE was prepared from rat whole blood and the AChE activity was quantified (0.16 U/mL). The inhibition ability of FNT and FNO towards rhAChE and rat erythrocyte AChE was evaluated using a modified protocol (Zhao et al. 2021), and assays were conducted in triplicate. Briefly, series of increasing concentrations of FNT or FNO in ethanol, 5000 μM chlorpyrifos-oxon in ethanol (CPO, positive control) and 100% ethanol (solvent control) were all diluted 50× in 100 mM sodium phosphate (pH 7.4, containing 0.1 mg/mL BSA). The incubation mixtures with a total volume of 50 μL were incubated in a 96 well-plate and consisted of 44 μL sodium phosphate (pH 7.4), and 5 μL FNO solution (final concentrations ranging from 0.005 to 5 μM), or 5 μL FNT solution (final concentrations ranging from 0.5 to 500 μM), or 5 μL positive control (CPO at a final concentration of 10 μM), or 5 μL solvent control (ethanol at a final concentration of 0.2%). To initiate the inhibition reaction, 1 μL rhAChE (0.16 U/mL) or self-prepared rat erythrocyte AChE (0.16 U/mL) was added into each well. After 15 min incubation at 37℃, 150 μL reaction reagent (mixture of ATC at a final concentration of 150 μM and DTNB at a final concentration of 75 μM) was added, and the absorbance at 412 nm was measured continuously for 10 min at 37℃ to test the remaining AChE activity. The AChE activity was expressed as the remaining AChE activity relative to solvent control (100% activity) and positive control (0% activity) based on the equation (Eq S2):

$$\text{AChE activity\%} = \frac{A_{412}(t_{10} - t_0)\ \text{Test Compound} - A_{412}(t_{10} - t_0)\ \text{Positive Control}}{A_{412}(t_{10} - t_0)\ \text{Solvent Control} - A_{412}(t_{10} - t_0)\ \text{Positive Control}} \times 100\% \qquad \text{(Eq S2)}$$

where $A_{412}(t_{10} - t_0)$ *Test Compound* is the change of the absorbance at 412 nm between 0 min and 10 min for the test compound; similarly, $A_{412}(t_{10} - t_0)$ *Positive Control* is the change of the absorbance for the CPO sample, and $A_{412}(t_{10} - t_0)$ *Solvent Control* the change of the absorbance for the 0.2% ethanol sample.

Sensitivity analysis

A local sensitivity analysis was performed to identify the influential parameters on model outputs. In the current study, the maximum blood FNO concentration was used as the model output, considering that FNO is a more potent AChE inhibitor compared to its precursor FNT and its internal concentration is relevant for the toxicity prediction following acute FNT exposure. The normalized sensitivity coefficients (SCs) were calculated with the equation (Eq S3)

$$\text{SC} = \frac{(C' - C)}{(P' - P)} \times \frac{P}{C} \qquad \text{(Eq S3)}$$

where P represents the original parameter value in the PBK model and P' is the parameter value with a 5% increase, C is the model output with the initial parameter values and C' is the model output with a parameter value after a 5% increase. Parameters with an absolute SC greater than 0.1 were considered to have a substantial influence on the model output (WHO 2010). The sensitivity analysis was carried out using oral dose levels of 0.25 mg/kg BW and 0.33 mg/kg BW for rats and humans, respectively, representing the rat and human NOAELs derived by US EPA (2010) and APVMA (2023).

BMD analysis

BMD modeling was used to derive POD values from the predicted *in vivo* dose-response curves for rats and humans. The EFSA web-tool (https://efsa.openanalytics.eu/) integrated with the R package PROAST (version 70.0) developed by the Dutch National Institute for Public Health and the Environment (RIVM) was used for the BMD analysis. A BMD value resulting in a 10% benchmark response (BMR) change with lower 95% confidence limit was defined as $BMDL_{10}$. Briefly, the continuous data were fitted to a set of models (Exponential, Hill, Inverse Exponential, and Log-Normal family models), and all fitted models excluding the FULL and NULL models were used for model averaging via a weighted average model. More weight was given to the models with lower Akaike's Information Criterion (AIC), and an averaged confidence interval was estimated using the recommended defaults.

Table S1 Summary of physiological and physicochemical parameters used for the rat and human PBK models.

Model parameters	Symbols in model code	Rat	Human
Physiological parameters			
Body weight (kg)	BW	0.25	70
Percentage of body weight [a]			
Liver	VLc	3.4	2.6
Fat	VFc	7.0	21.4
Kidney	VKc	0.7	0.4
Rapidly perfused tissue	VRc	9.1	6.4
Slowly perfused tissue	VSc	72.4	61.3
Blood	VB	7.4	7.9
Flow (L/hr/$BW^{0.74}$) [a]			
Cardiac output	QC	15.0	15.0
Percentage of cardiac output [a]			
Liver	QLc	25.0	22.7
Fat	QFc	9.0	5.2
Kidney	QKc	20	17.5
Rapidly perfused tissue	QRc	31.6	19.5
Slowly perfused tissue	QSc	14.4	35.1
Tissue: blood partition coefficients for FNT [b]			
Liver	PLFNT	4.02	5.85
Fat	PFFNT	13.58	12.58
Kidney	PKFNT	3.78	3.78
Rapidly perfused tissue	PRFNT	5.78	6.55
Slowly perfused tissue	PSFNT	3.45	5.28
Tissue: blood partition coefficients for FNO [b]			
Liver	PLFNO	3.60	5.29
Fat	PFFNO	11.22	10.44
Kidney	PKFNO	3.40	3.45
Rapidly perfused tissue	PRFNO	5.13	5.90
Slowly perfused tissue	PSFNO	3.10	4.77
Tissue: blood partition coefficients for MNP [b]			
Liver	PLMNP	0.29	0.29
Fat	PFMNP	0.15	0.15
Kidney	PKMNP	0.36	0.36
Rapidly perfused tissue	PRMNP	0.45	0.40
Slowly perfused tissue	PSMNP	0.38	0.28

a: obtained from Brown et al. (1997) and Gearhart et al. (1990);
b: obtained by dividing tissue:plasma partition coefficients (predicted by QIVIVE tools, version 2.0, Punt et al. 2021) by the blood/plasma ratio.

Table S2 Summary of LogP and pKa values of FNT, FNO and MNP. Values used in the current study are presented in bold.

Parameter	Source	FNT	FNO	MNP
LogP	Experimental data	3.319 (JMPR 2003)	NA*	NA
	Reported data (without specific information to judge if the value is experimental data or *in-silico* prediction)	3.16 (WHO 2004)<br>3.3 (Pehkonen and Zhang. 2002)<br>3.43 (Story et al. 2012)	NA	2.12 (OECD 1994)
	In silico predicted data	**2.799** (Pan et al. 2021; Pires et al. 2015)<br>3.115 (Chemicalize)<br>3.3 (PubChem)	1.7 (PubChem)<br>2.225 (Chemicalize)<br>**2.683** (Pan et al. 2021; Pires et al. 2015)	**1.609** (Pan et al. 2021; Pires et al. 2015)<br>2.123 (Chemicalize)<br>2.5 (PubChem)
pKa	Experimental data	NA	NA	NA
	Reported data (without specific information to judge if the value is experimental data or *in-silico* prediction)	Not applicable (JMPR 2003)	NA	Not applicable (OECD 1994)<br>7.33 (Shahpoury et al. 2018)
	In silico predicted data	No ionizable atoms found (Chemicalize; Pan et al. 2021)	No ionizable atoms found (Chemicalize; Pan et al. 2021)	**6.9** (Pan et al. 2021)<br>7.33 (Chemicalize)

*: not available.

Table S3 Summary of available *in vivo* kinetic studies in rats and humans following single FNT exposure.

Species	FNT dose	Exposure route	Available data	Reference
Rat (male and female, HLA-Wistar rat, 6-week-old, 180-200 g)	15 mg/kg BW	Single oral dose (gavage), 5% Sorpol:xylene (1:2) as vehicle, free access to water and diet	Time-dependent blood FNT concentration	Miyamoto et al. 1976
Rat (male, HLA-Wistar rat, 13-weed-old, body weight not provided)	15 and 50 mg/kg BW	Single oral dose (gavage), 10% Tween 80 as vehicle	Time-dependent blood FNT concentration	Miyamoto et al. 1977
Rat (female, albino rat, 180-200 g, age not provided)	47 mg/kg BW	Single oral dose (gavage), edible oil as vehicle	Time-dependent cumulative urinary MNP	Hladká and Nosáľ. 1967
	0.94 mg/kg BW	Single intravenous dose, eryfor and water as vehicle	Time-dependent cumulative urinary MNP	
Human (12 volunteers, 8 males and 4 females, mean age 33 years)	0.09 and 0.18 mg/kg BW	Single oral dose for the first 12 hours, unknown vehicle, in capsule given with food	Time-dependent blood FNT concentration*	Meaklim et al. 2003
Human (24 volunteers, gender and age not provided)	0.042, 0.083, 0.17, 0.25 and 0.33 mg/kg BW	Single oral dose, olive oil as vehicle, in gelatine capsule	Dose-dependent cumulative urinary MNP excreting within 24 hours	Nosáľ and Hladká. 1968

*: in the reference, it mentioned in the text that "whole-blood concentrations of fenitrothion" were shown in Table 1 and 2, while in the captions of Table 1 and 2, it was written as "fenitrothion plasma concentrations". We used the data in Table 1 and 2 as blood concentrations of fenitrothion (FNT) for the model evaluation, considering that the extraction and quantification were based on the whole blood samples as described in the "Analysis" section (Materials and Methods, Meaklim et al. 2003).

Table S4 Summary of *in vitro* kinetic parameters for the biotransformation of FNT and FNO.

Kinetic parameter	Rat	Human
**Liver**		
**FNT to FNO** (CYP450s-catalyzed)		
V_{max} (nmol/min/mg microsomal protein)	4.11	1.68
K_m (μM)	8.28	21.56
Catalytic efficiency (μL/min/mg protein)*	496.38	77.92
Scaled V_{max} (μmol/hr)	73.42	5870.59
**FNT to MNP** (CYP450s-catalyzed)		
V_{max} (nmol/min/mg microsomal protein)	0.76	0.18
K_m (μM)	20.51	4.08
Catalytic efficiency (μL/min/mg protein)*	37.06	44.12
Scaled V_{max} (μmol/hr)	13.59	637.73
**FNO to MNP** (PON1-catalyzed)		
V_{max} (nmol/min/mg microsomal protein)	25.38	3.09
K_m (μM)	4528	12564
Catalytic efficiency (μL/min/mg protein)*	5.61	0.25
Scaled V_{max} (μmol/hr)	453.03	10794.20
**Plasma**		
**FNO to MNP** (PON1-catalyzed)		
V_{max} (nmol/min/mg plasma protein)	19.26	2.14
K_m (μM)	4075	5489
Catalytic efficiency (μL/min/mg protein)*	4.73	0.39
Scaled V_{max} (μmol/hr)	693.74	25798.98

*: calculated as V_{max}/K_m.

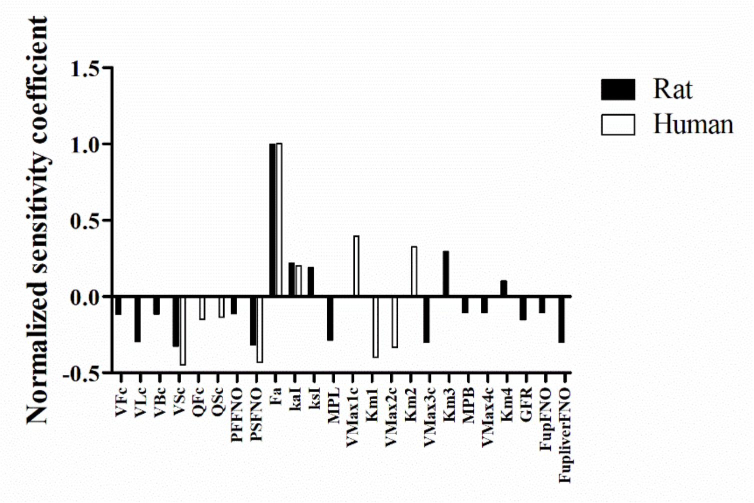

Figure S1 Results of a local sensitivity analysis for the predicted maximum blood FNO concentration at dose levels of 0.25 mg/kg BW (rats) and 0.33 mg/kg BW (humans). Model parameters with a normalized SC with an absolute value higher than 0.1 are shown. VFc, fraction of fat tissue; VLc, fraction of liver tissue; VBc, fraction of blood; VSc, fraction of slowly perfused tissue; QFc, fraction of blood flow to fat tissue; QSc, fraction of blood flow to slowly perfused tissue; PFFNO, fat:blood partition coefficient of FNO; PSFNO, slowly perfused tissue:blood partition coefficient of FNO; Fa, fraction of dose absorbed; kaI, absorption rate constant from intestine to liver; ksI, transfer rate constant from stomach to intestine; MPL, liver microsomal protein yield scaling factor; Vmax1c, maximum rate for conversion of FNT to FNO; Km1, Michaelis-Menten constant for conversion of FNT to FNO; Vmax2c, maximum rate for conversion of FNT to MNP; Km2, Michaelis-Menten constant for conversion of FNT to MNP; Vmax3c, maximum rate for conversion of FNO to MNP in liver; Km3, Michaelis-Menten constant for conversion of FNO to MNP in liver; MPB, plasma protein concentration; Vmax4c, maximum rate for conversion of FNO to MNP in plasma; Km4, Michaelis-Menten constant for conversion of FNO to MNP in plasma; GFR, glomerular filtration rate; FupFNO, unbound fraction of FNO in plasma; FupliverFNO, adjusted unbound fraction of FNO in plasma for hepatic biotransformation.

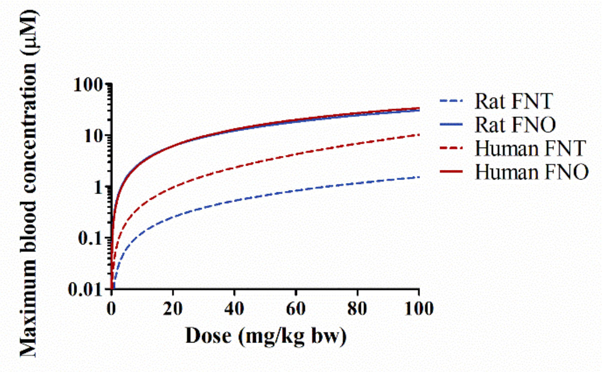

Figure S2 PBK modeling-based predictions of maximum blood FNT and FNO concentrations in rats and humans under increasing FNT dose levels (0.01 – 100 mg/kg BW).

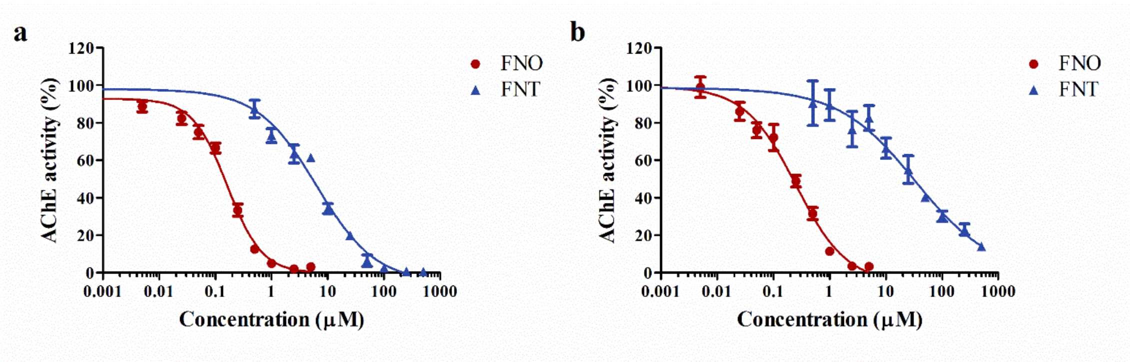

Figure S3 Activity of (a) self-prepared rat erythrocyte AChE and (b) recombinant human AChE upon incubation with increasing FNT and FNO concentrations. Results are presented as means ± SEM from three independent experiments. The IC_{50} values of FNT are 8.24 and 30.93 μM for rats and humans, and the IC_{50} values of FNO are 0.18 and 0.25 μM for rats and humans, respectively.

Results of BMD analysis for rats

Results from a BMD analysis for the predicted *in vivo* dose-response curve for rat erythrocyte AChE inhibition upon acute oral FNT exposure (Figure 7a). The table and figures present the characteristics of fitted models, the weights for model averaging and the final benchmark dose for 10% effect with the 95% lower–upper confidence limit values of the benchmark dose (BMDL-BMDU).

model	converged	loglik	npar	AIC	Weights	Final BMD values (mg/kg BW)	
full model	yes	37.38	12	-50.76	-	$BMDL_{10}$	$BMDU_{10}$
null model	yes	-49.32	2	102.64	-		
Expon. m3-	yes	-16.16	4	40.32	-		
Expon. m5-	yes	34.67	5	-59.34	0.19		
Hill m3-	yes	-16.05	4	40.10	-		
Hill m5-	yes	36.10	5	-62.20	0.81	1.30	1.71
Inv.Expon. m3-	yes	-14.90	4	37.80	-		
Inv.Expon. m5-	yes	-4.92	5	19.84	0		
LN m3-	yes	-15.55	4	39.10	-		
LN m5-	yes	10.32	5	-10.64	0		

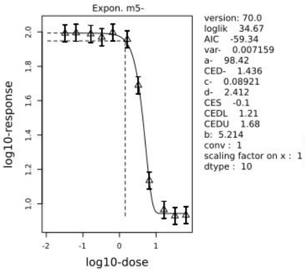

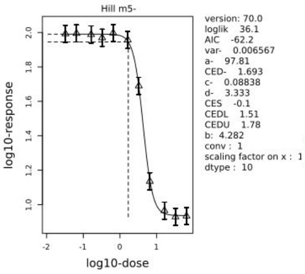

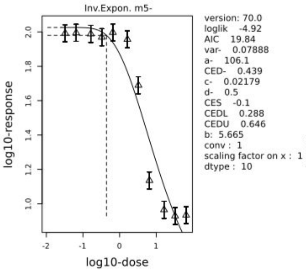

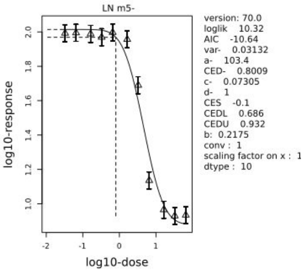

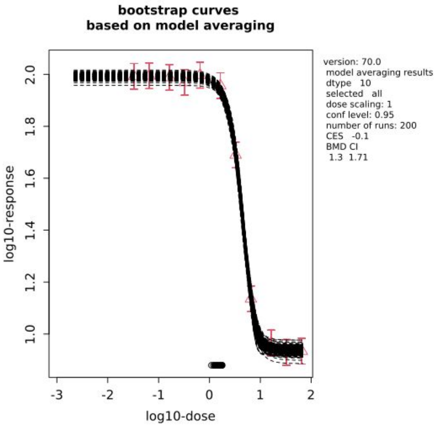

Results of BMD analysis for humans

Results from a BMD analysis for the predicted *in vivo* dose-response curve for human erythrocyte AChE inhibition upon acute oral FNT exposure (Figure 7b). The table and figures present the characteristics of fitted models, the weights for model averaging and the final benchmark dose for 10% effect with the 95% lower–upper confidence limit values of the benchmark dose (BMDL-BMDU).

model	converged	loglik	npar	AIC	Weights	Final BMD values (mg/kg BW)	
full model	yes	50.00	12	-76.00	-	$BMDL_{10}$	$BMDU_{10}$
null model	yes	-41.37	2	86.74	-		
Expon. m3-	yes	10.21	4	-12.42	-		
Expon. m5-	yes	38.52	5	-67.04	0.98		
Hill m3-	yes	10.40	4	-12.80	-		
Hill m5-	yes	34.51	5	-59.02	0.02	0.26	0.66
Inv.Expon. m3-	yes	12.69	4	-17.38	-		
Inv.Expon. m5-	yes	26.32	5	-42.64	0		
LN m3-	yes	11.46	4	-14.92	-		
LN m5-	yes	32.59	5	-55.18	0		

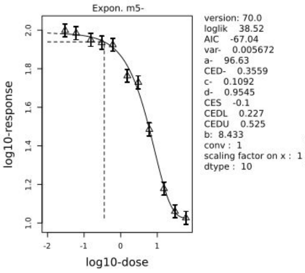

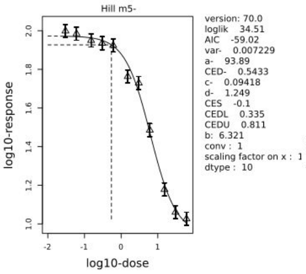

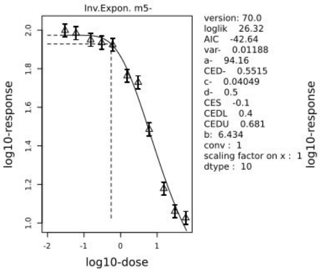

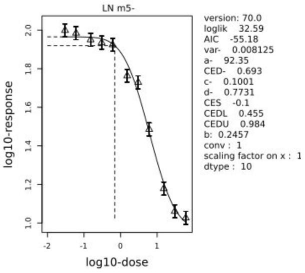

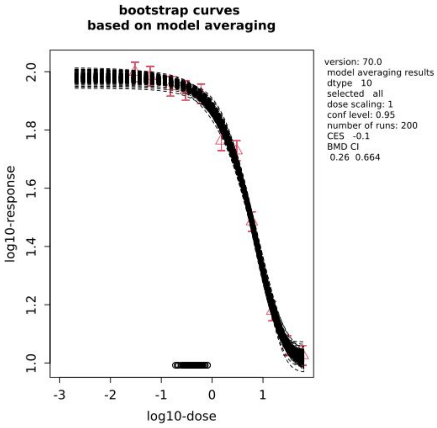

PBK model code

Rat model

```
;Date: August 22, 2023
;Purpose: PBK model Fenitrothion, built with in silico and in vitro derived parameter values
;Species: Rat, mixed gender
;Complied by: Jiaqi Chen and Shensheng Zhao
;Organization: Wageningen University and Research

;==========================================================================
;Physiological parameters
;==========================================================================
;tissue volume (Brown et al. 1997)
BW=0.25                  ; (kg), body weight rat
VFc=0.070                ; fraction of fat tissue
VLc=0.034                ; fraction of liver tissue
VKc= 0.007               ; fraction of kidney tissue
VBc=0.074                ; fraction of blood
VRc=0.091                ; fraction of rapidly perfused tissue
VSc=0.724                ; fraction of slowly perfused tissue
                         ; total fraction is 1
VF = VFc*BW              ; (L or kg), volume of fat tissue (calculated)
VL = VLc*BW              ; (L or kg), volume of liver tissue (calculated)
VK = VKc*BW              ; (L or kg), volume of kidney tissue (calculated)
VB = VBc*BW              ; (L or kg), volume of blood (calculated)
VR = VRc*BW              ; (L or kg), volume of rapidly perfused tissue (calculated)
VS = VSc*BW              ; (L or kg), volume of slowly perfused tissue (calculated)
;------------------------------------------------------------------------------------------------------------------
;blood flow rates (Gearhart et al. 1990)
QC = 15*BW^0.74          ; (L/hr), cardiac output (Brown et al. 1997)
QFc = 0.090              ; fraction of blood flow to fat
QLc = 0.250              ; fraction of blood flow to liver
QKc = 0.200              ; fraction of blood flow to kidney
QRc = 0.316              ; fraction of blood flow to rapidly perfused tissue
QSc = 0.144              ; fraction of blood flow to slowly perfused tissue
                         ; total fraction is 1
QF = QFc*QC              ; (L/hr), blood flow to fat tissue (calculated)
QL = QLc*QC              ; (L/hr), blood flow to liver tissue (calculated)
QK =QKc*QC               ; (L/hr), blood flow to kidney tissue (calculated)
QR = QRc*QC              ; (L/hr), blood flow to rapidly perfused tissue (calculated)
QS = QSc*QC              ; (L/hr), blood flow to slowly perfused tissue (calculated)

;=====================================================================
;Physicochemical parameters
;=====================================================================
;tissue:blood partition coefficients were obtained by dividing tissue:plasma partition coefficients (QIVIVE tools, version 2.0, Punt et al. 2021) by the corresponding blood/plasma ratio (BPr), which is assumed to be 0.55 for neutral (Fenitrothion and Fenitrooxon) and acidic compounds (3-Methyl-4-nitrophenol) (Badhan et al. 2014).

;partition coefficients Fenitrothion (FNT) (tissue : blood)
;LogP (FNT) = 2.799      ; MolGpka (Pan et al. 2021)
;pKa (FNT): n/a          ; MolGpka (Pan et al. 2021)
PFFNT = 13.58            ; fat/blood partition coefficient of Fenitrothion
PLFNT = 4.02             ; liver/blood partition coefficient of Fenitrothion
PKFNT = 3.78             ; kidney/blood partition coefficient of Fenitrothion
PRFNT = 5.78             ; rapidly perfused tissue/blood partition coefficient of Fenitrothion
PSFNT = 3.45             ; slowly perfused tissue/blood partition coefficient of Fenitrothion
;-----------------------------------------------------------------------------------------------------------
;partition coefficients Fenitrooxon (FNO) (tissue : blood)
;LogP (FNO) = 2.683      ; MolGpka (Pan et al. 2021)
;pKa (FNO): n/a          ; MolGpka (Pan et al. 2021)
PFFNO = 11.22            ; fat/blood partition coefficient of Fenitrooxon
PLFNO = 3.60             ; liver/blood partition coefficient of Fenitrooxon
PKFNO = 3.40             ; kidney/blood partition coefficient of Fenitrooxon
PRFNO = 5.13             ; rapidly perfused tissue/blood partition coefficient of Fenitrooxon
PSFNO = 3.10             ; slowly perfused tissue/blood partition coefficient of Fenitrooxon
;-----------------------------------------------------------------------------------------------------------
;partition coefficients 3-Methyl-4-nitrophenol (MNP) (tissue : blood)
;LogP (MNP) = 1.609      ; MolGpka (Pan et al. 2021)
```

```
;pKa (MNP) = 6.9              ; MolGpka (Pan et al. 2021)
PFMNP = 0.15                  ; fat/blood partition coefficient of 3-Methyl-4-nitrophenol
PLMNP = 0.29                  ; liver/blood partition coefficient of 3-Methyl-4-nitrophenol
PKMNP = 0.36                  ; kidney/blood partition coefficient of 3-Methyl-4-nitrophenol
PRMNP = 0.45                  ; rapidly perfused tissue/blood partition coefficient of 3-Methyl-4-nitrophenol
PSMNP = 0.38                  ; slowly perfused tissue/blood partition coefficient of 3-Methyl-4-nitrophenol

;=====================================================================
;Kinetic parameters
;=====================================================================
;Transport from needle to blood
kn  =  1000000        ; (/hr), injection
;---------------------------------------------------------------------------------------------------------------
;Fa=Fraction absorbed
Fa = 0.9              ; EFSA 2006
;Absorption/transfer rate constant
kaS  = 0.1            ; a value set equal to that for diazinon, Zhao et al, 2021 (stomach; /hr)
kaI = 0.59            ; a value set equal to that for diazinon, Zhao et al, 2021 (intestine; /hr)
ksI = 0.48            ; a value set equal to that for diazinon, Zhao et al, 2021 (transfer stomach-intestine; /hr)
;---------------------------------------------------------------------------------------------------------------
;Metabolism liver
MPL=35  ; scaling factor of rat liver microsome (mg microsomal protein /g liver) (Medinsky et al. 1994)
;based on metabolite formation from FNT to FNO, scaled maximum rate of metabolism (umol/hr)
VMax1c =  4.113 {nmol/min/mg}             ; (FNT-->FNO, CYP450), data derived from experiment
VMax1=VMax1c/1000*60*MPL*1000*VL          ; 1000-->nmol to umol
                                          ; 60-->min to hour
                                          ; MPL-->mg microsomal protein /g liver
                                          ; 1000-->kg to g  (liver)
                                          ; VL-->volume of liver tissue {kg} (VLc*BW)
;affinity constants (umol/L)
Km1 = 8.28            ; data derived from experiment
;---------------------------------------------------------------------------------------------------------------
;Metabolism liver
;based on metabolite formation from FNT to MNP, scaled maximum rate of metabolism (umol/hr)
VMax2c =  0.7612 {nmol/min/mg}            ; (FNT-->MNP, CYP450), data derived from experiment
VMax2=VMax2c/1000*60*MPL*1000 *VL         ; 1000-->nmol to umol
                                          ; 60-->min to hour
                                          ; MPL-->mg microsomal protein /g liver
                                          ; 1000-->kg to g  (liver)
                                          ; VL-->volume of liver tissue {kg} (VLc*BW)
;affinity constants (umol/L)
Km2 = 20.51           ; data derived from experiment
;---------------------------------------------------------------------------------------------------------------
;Metabolism liver
;based on metabolite formation from FNO to MNP, scaled maximum rate of metabolism (umol/hr)
VMax3c= 25.38 {nmol/min/mg}                            ; (FNO-->MNP, PON1), data derived from experiment
VMax3 = VMax3c/1000*60*MPL*1000*VL {umol/hr/liver}     ; 1000-->nmol to umol
                                                       ; 60-->min to hour
                                                       ; MPL-->mg microsomal protein /g liver
                                                       ; 1000-->kg to g  (liver)
                                                       ; VL-->volume of liver tissue {kg} (VLc*BW)
;affinity constants (umol/L)
Km3 = 4528            ; data derived from experiment
;---------------------------------------------------------------------------------------------------------------
;Metabolism blood
MPB= 59  ; scaling factor of plasma protein (mg plasma protein / mL plasma-->total protein concentration of plasma), data obtained
from experiment
;based on metabolite formation from FNO to MNP, scaled maximum rate of metabolism (umol/hr)
VMax4c=19.26 {nmol/min/mg plasma protein}                   ; (FNO-->MNP, PON1), data derived from experiment
VMax4 = VMax4c/1000*60*MPB*1000*VB*0.55 {umol/hr/plasma}    ; 1000-->nmol to umol
                                                            ; 60-->min to hour
                                                            ; MPB-->mg protein /mL plasma
                                                            ; 1000-->L to mL  (blood)
                                                            ; VB-->volume of blood {L} (VBc*BW)
                                                            ; 0.55-->plasma makes up ~55% of total blood, data
                                                            derived from https:www.blood.co.uk/why-give-
                                                            blood/how-blood-is-used/blood-components/plasma/,
                                                            since the PON1 is mainly exist in plasma, thus we assume
                                                            metabolism in plasma is equal to metabolism in blood.
;affinity constants (umol/L)
Km4 = 4075            ; data derived from experiment
```

```
;-------------------------------------------------------------------------------------------------------------------
;Excretion from kidney via glomerular filtration
GFR = 5.2                                ; (mL/min/kg bw), Walton et al., 2004
GF = GFR/1000*BW*60                      ; (L/hr), rat glomerular filtration rate

;Fraction unbound, predicted by QIVIVE tools (Punt et al. 2021)
FupFNO = 0.158                ; Fraction unbound in plasma (Fup) for FNO
FupliverFNT = 0.643           ; Adjusted Fup for FNT for hepatic metabolism (Poulin and Haddad. 2021)
FupliverFNO = 0.670           ; Adjusted Fup for FNO for hepatic metabolism (Poulin and Haddad. 2021)

;==================================================================
;Run settings
;==================================================================
;Molecular weight
MWFNT = 277.23                ; Molecular weight Fenitrothion (FNT)
MWFNO = 261.17                ; Molecular weight Fenitrooxon (FNO)
MWMNP = 153.14                ; Molecular weight 3-Methyl-4-nitrophenol (MNP)

;Oral dose
ODOSEmg = 15                                      ; given oral dose in mg/kg bw
ODOSEumol2 = ODOSEmg*1E-3/MWFNT*1E6               ; given oral dose recalculated to umol/kg bw
ODOSEumol=ODOSEumol2*BW                           ; given oral dose in umol
;-------------------------------------------------------------------------------------------------------------------
;IV dose
IVDOSEmg = 0                                      ; given IV dose in mg/kg bw
IVDOSEumol2 = IVDOSEmg*1E-3/MWFNT*1E6             ; given IV dose recalculated to umol/kg bw
IVDOSEumol=IVDOSEumol2*BW                         ; umol given IV

;Time
Starttime = 0        ; in hr
Stoptime = 24        ; in hr

;==================================================================
;Model calculations
;Fenitrothion (FNT) Model
;==================================================================
;needle compartment
;ANe  = amount in needle, umol
ANe'  = -kn*ANe
Init ANe = IVDOSEumol
;-------------------------------------------------------------------------------------------------------------
;stomach compartment
;Ast1  = amount of FNT remaining in stomach tissue (umol)
Ast1'  = -kaS*Ast1-ksI*Ast1
Init Ast1 =ODOSEumol*Fa
;-------------------------------------------------------------------------------------------------------------
;intestine compartment
;Ast2  = amount of FNT remaining in intestine tissue (umol)
Ast2' =ksI*Ast1-kaI*Ast2
Init Ast2 =0
;-------------------------------------------------------------------------------------------------------------------
;liver compartment
;ALFNT = Amount FNT in liver tissue (umol)
ALFNT' = kaS*Ast1 + kaI*Ast2 + QL*(CBFNT - CVLFNT) - AMFO'- AMFT1'
Init ALFNT = 0
CLFNT = ALFNT/VL
CVLFNT = CLFNT/PLFNT

;AMFO = amount FNT metabolized to the metabolite FNO
AMFO' =VMax1*CVLFNT*(FupliverFNT/0.55)/(Km1 + CVLFNT*(FupliverFNT/0.55))
Init AMFO = 0

;AMFT1 = amount FNT metabolized to the metabolite MNP (and DMTP)
AMFT1' = VMax2*CVLFNT*(FupliverFNT/0.55)/(Km2 + CVLFNT*(FupliverFNT/0.55))
Init AMFT1 = 0
; 0.55 is the BPr value for FNT as a neutral compound, which is used here to correct the adjusted unbound fraction in liver plasma
(Fupliver) to unbound fraction in blood
;-------------------------------------------------------------------------------------------------------------------
;kidney compartment
;AKFNT = Amount FNT in kidney tissue (umol)
AKFNT' = QK*(CBFNT-CVKFNT) - AFNTexe'
```

```
Init AKFNT = 0
CKFNT = AKFNT/VK
CVKFNT = CKFNT/PKFNT

;AFNTexe = Amount FNT eliminated to urine (umol)
AFNTexe' = GF * CVKFNT
Init AFNTexe = 0
;-----------------------------------------------------------------------------------------------------------------------
;fat compartment
;AFFNT = Amount FNT in fat tissue (umol)
AFFNT' = QF*(CBFNT-CVFFNT)
Init AFFNT = 0
CFFNT = AFFNT/VF
CVFFNT = CFFNT/PFFNT
;-----------------------------------------------------------------------------------------------------------------------
;tissue compartment (rapidly perfused tissue)
;ARFNT = Amount FNT in rapidly perfused tissue (umol)
ARFNT' = QR*(CBFNT-CVRFNT)
Init ARFNT = 0
CRFNT = ARFNT/VR
CVRFNT = CRFNT/PRFNT
;-----------------------------------------------------------------------------------------------------------------------
;tissue compartment (slowly perfused tissue)
;ASFNT = Amount FNT in slowly perfused tissue (umol)
ASFNT' = QS*(CBFNT-CVSFNT)
Init ASFNT = 0
CSFNT = ASFNT/VS
CVSFNT = CSFNT/PSFNT
;-----------------------------------------------------------------------------------------------------------------------
;blood compartment
;ABFNT = Amount FNT in blood (umol)
ABFNT' = kn*ANe +QF*CVFFNT +QL*CVLFNT +QK*CVKFNT  +QS*CVSFNT +QR*CVRFNT -
(QF+QL+QK+QS+QR)*CBFNT
Init ABFNT = 0
CBFNT = ABFNT/VB

;=========================================================================
;Fenitrooxon (FNO) submodel
;=========================================================================
;liver compartment
;ALFNO = Amount FNO in liver tissue (umol)
ALFNO' =  QL*(CBFNO - CVLFNO) +AMFO' - AMFT2'
Init ALFNO = 0
CLFNO = ALFNO/VL
CVLFNO = CLFNO/PLFNO

;AMFT2 = Amount FNO metabolized to the metabolite MNP (and DMP)
AMFT2' = VMax3*CVLFNO*(FupliverFNO/0.55)/(Km3 + CVLFNO*(FupliverFNO/0.55))
Init AMFT2  = 0
; 0.55 is the BPr value for FNO as a neutral compound, which is used here to correct the adjusted unbound fraction in liver plasma
(Fupliver) to unbound fraction in blood
;-----------------------------------------------------------------------------------------------------------------------
;kidney compartment
;AKFNO = Amount FNO in kidney tissue (umol)
AKFNO' = QK*(CBFNO-CVKFNO) - AFNOexe'
Init AKFNO = 0
CKFNO = AKFNO/VK
CVKFNO = CKFNO/PKFNO

;AFNOexe = Amount FNO eliminated to urine (umol)
AFNOexe' = GF * CVKFNO
Init AFNOexe = 0
;-----------------------------------------------------------------------------------------------------------------------
;fat compartment
;AFFNO = Amount FNO in fat tissue (umol)
AFFNO' = QF*(CBFNO-CVFFNO)
Init AFFNO = 0
CFFNO = AFFNO/VF
CVFFNO = CFFNO/PFFNO
 ;-----------------------------------------------------------------------------------------------------------------------
;tissue compartment (rapidly perfused tissue)
```

```
;ARFNO = Amount FNO in rapidly perfused tissue (umol)
ARFNO' = QR*(CBFNO-CVRFNO)
Init ARFNO = 0
CRFNO = ARFNO/VR
CVRFNO = CRFNO/PRFNO
;------------------------------------------------------------------------------------------------------------------
;tissue compartment (slowly perfused tissue)
;ASFNO = Amount of FNO in slowly perfused tissue (umol)
ASFNO' = QS*(CBFNO-CVSFNO)
Init ASFNO = 0
CSFNO = ASFNO/VS
CVSFNO = CSFNO/PSFNO
;------------------------------------------------------------------------------------------------------------------
;blood compartment
;ABFNO = Amount FNO in blood (umol)
ABFNO' = QF*CVFFNO +QL*CVLFNO +QK*CVKFNO +QS*CVSFNO +QR*CVRFNO -(QF+QL+QK+QS+QR)*CBFNO -
AMFT3'
Init ABFNO = 0
CBFNO = ABFNO/VB

;AMFT3 = amount FNO metabolized to the metabolite MNP (and DMP)
AMFT3' = VMax4*CBFNO*(FupFNO/0.55)/(Km4 + CBFNO*(FupFNO/0.55))
Init AMFT3  = 0
; 0.55 is the BPr value for FNO as a neutral compound, which is used here to correct the unbound fraction in plasma (Fup) to
unbound fraction in blood

;=========================================================================
;3-Methyl-4-nitrophenol (MNP) submodel
;=========================================================================
;liver compartment
;ALMNP = Amount MNP in liver tissue (umol)
ALMNP' =  QL*(CBMNP - CVLMNP) +AMFT1' +AMFT2'
Init ALMNP = 0
CLMNP = ALMNP/VL
CVLMNP = CLMNP/PLMNP
;------------------------------------------------------------------------------------------------------------------
;kidney compartment
;AKMNP = Amount MNP in kidney tissue (umol)
AKMNP' = QK*(CBMNP-CVKMNP) - AMNPexe'
Init AKMNP = 0
CKMNP = AKMNP/VK
CVKMNP = CKMNP/PKMNP

;AMNPexe = Amount MNP eliminated to urine (umol)
AMNPexe' = GF * CVKMNP
Init AMNPexe = 0
;------------------------------------------------------------------------------------------------------------------
;fat compartment
;AFMNP = Amount MNP in fat tissue (umol)
AFMNP' = QF*(CBMNP-CVFMNP)
Init AFMNP = 0
CFMNP = AFMNP/VF
CVFMNP = CFMNP/PFMNP
 ;------------------------------------------------------------------------------------------------------------------
;tissue compartment (rapidly perfused tissue)
;ARMNP = Amount MNP in rapidly perfused tissue (umol)
ARMNP' = QR*(CBMNP-CVRMNP)
Init ARMNP = 0
CRMNP = ARMNP/VR
CVRMNP = CRMNP/PRMNP
;------------------------------------------------------------------------------------------------------------------
;tissue compartment (slowly perfused tissue)
;ASMNP = Amount MNP in slowly perfused tissue (umol)
ASMNP' = QS*(CBMNP-CVSMNP)
Init ASMNP = 0
CSMNP = ASMNP/VS
CVSMNP = CSMNP/PSMNP
;------------------------------------------------------------------------------------------------------------------
; blood compartment
;ABMNP = Amount MNP in blood (umol)
```

```
ABMNP' = QF*CVFMNP +QL*CVLMNP  +QK*CVKMNP +QS*CVSMNP +QR*CVRMNP +AMFT3'-
(QF+QL+QK+QS+QR)*CBMNP
Init ABMNP = 0
CBMNP = ABMNP/VB

;=========================================================================
;Mass balance calculations for FNT model
TotalFNT = ODOSEumol *Fa+ IVDOSEumol
CalculatedFNT = ANe +Ast1 +Ast2 +ALFNT +AKFNT +AFFNT +ASFNT +ARFNT +ABFNT+AMFO +AMFT1 +AFNTexe
ERRORFNT=(TotalFNT-CalculatedFNT)/(TotalFNT+1E-30)*100
MASSBBALFNT=TotalFNT-CalculatedFNT + 1

;=========================================================================
;Mass balance calculations for FNO sub-model
TotalFNO = AMFO
CalculatedFNO = ALFNO +AKFNO +AFFNO +ASFNO +ARFNO +ABFNO +AMFT2 +AMFT3 +AFNOexe
ERRORFNO=(TotalFNO-CalculatedFNO)/(TotalFNO+1E-30)*100
MASSBBALFNO=TotalFNO-CalculatedFNO + 1

;=========================================================================
;Mass balance calculations for MNP sub-model
TotalMNP = AMFT1 +AMFT2 +AMFT3
CalculatedMNP = ALMNP +AKMNP +AFMNP +ASMNP +ARMNP +ABMNP +AMNPexe
ERRORMNP=(TotalMNP-CalculatedMNP)/(TotalMNP+1E-30)*100
MASSBBALMNP=TotalMNP-CalculatedMNP + 1
```

Human model

;Date: August 22, 2023
;Purpose: PBK model Fenitrothion, built with *in silico* and *in vitro* derived parameter values
;Species: Human, mixed gender
;Complied by: Jiaqi Chen and Shensheng Zhao
;Organization: Wageningen University and Research

```
;=============================================================================
;Physiological parameters
;=============================================================================
;tissue volume (Brown et al. 1997)
BW=70                  ; (kg), body weight
VFc=0.214              ; fraction of fat tissue
VLc=0.026              ; fraction of liver tissue
VKc=0.004              ; fraction of kidney tissue
VBc=0.079              ; fraction of blood
VRc=0.064              ; fraction of rapidly perfused tissue
VSc=0.613              ; fraction of slowly perfused tissue
                       ; total fraction is 1
VF = VFc*BW            ; (L or kg), volume of fat tissue (calculated)
VL = VLc*BW            ; (L or kg), volume of liver tissue (calculated)
VK = VKc*BW            ; (L or kg), volume of kidney tissue (calculated)
VB = VBc*BW            ; (L or kg), volume of blood (calculated)
VR = VRc*BW            ; (L or kg), volume of rapidly perfused tissue (calculated)
VS = VSc*BW            ; (L or kg), volume of slowly perfused tissue (calculated)
;-------------------------------------------------------------------------------------------------------------------
;blood flow rates (Brown et al. 1997)
QC = 15*BW^0.74        ; (L/hr), cardiac output
QFc = 0.052            ; fraction of blood flow to fat
QLc = 0.227            ; fraction of blood flow to liver
QKc = 0.175            ; fraction of blood flow to kidney
QRc = 0.195            ; fraction of blood flow to rapidly perfused tissue
QSc = 0.351            ; fraction of blood flow to slowly perfused tissue
                       ; total fraction is 1
QF = QFc*QC            ; (L/hr), blood flow to fat tissue (calculated)
QL = QLc*QC            ; (L/hr), blood flow to liver tissue (calculated)
QK =QKc*QC             ; (L/hr), blood flow to kidney tissue (calculated)
QR = QRc*QC            ; (L/hr), blood flow to rapidly perfused tissue (calculated)
QS = QSc*QC            ; (L/hr), blood flow to slowly perfused tissue (calculated)

;=======================================================================
;Physicochemical parameters
;=======================================================================
;tissue:blood partition coefficients were obtained by dividing tissue:plasma partition coefficients (QIVIVE tools, version 2.0, Punt et
al. 2021) by the corresponding blood/plasma ratio (BPr), which is assumed to be 0.55 for neutral (Fenitrothion and Fenitrooxon) and
acidic compounds (3-Methyl-4-nitrophenol) (Badhan et al. 2014).

;partition coefficients Fenitrothion (FNT) (tissue : blood)
;LogP (FNT) = 2.799     ; MolGpka (Pan et al. 2021)
;pKa (FNT): n/a         ; MolGpka (Pan et al. 2021)
PFFNT = 12.58           ; fat/blood partition coefficient of Fenitrothion
PLFNT = 5.85            ; liver/blood partition coefficient of Fenitrothion
PKFNT = 3.78            ; kidney/blood partition coefficient of Fenitrothion
PRFNT = 6.55            ; rapidly perfused tissue/blood partition coefficient of Fenitrothion
PSFNT= 5.28             ; slowly perfused tissue/blood partition coefficient of Fenitrothion
;-------------------------------------------------------------------------------------------------------------------
;partition coefficients Fenitrooxon (FNO) (tissue : blood)
;LogP (FNO) = 2.683     ; MolGpka (Pan et al. 2021)
;pKa (FNT): n/a         ; MolGpka (Pan et al. 2021)
PFFNO = 10.44           ; fat/blood partition coefficient of Fenitrooxon
PLFNO = 5.29            ; liver/blood partition coefficient of Fenitrooxon
PKFNO = 3.45            ; kidney/blood partition coefficient of Fenitrooxon
PRFNO = 5.90            ; rapidly perfused tissue/blood partition coefficient of Fenitrooxon
PSFNO = 4.77            ; slowly perfused tissue/blood partition coefficient of Fenitrooxon
;-------------------------------------------------------------------------------------------------------------------
;partition coefficients 3-Methyl-4-nitrophenol (MNP) (tissue : blood)
;LogP (MNP) = 1.609     ; MolGpka (Pan et al. 2021)
;pKa (MNP) = 6.9        ; MolGpka (Pan et al. 2021)
PFMNP = 0.15            ; fat/blood partition coefficient of 3-Methyl-4-nitrophenol
PLMNP = 0.29            ; liver/blood partition coefficient of 3-Methyl-4-nitrophenol
```

```
PKMNP = 0.36            ; kidney/blood partition coefficient of 3-Methyl-4-nitrophenol
PRMNP = 0.40            ; rapidly perfused tissue/blood partition coefficient of 3-Methyl-4-nitrophenol
PSMNP = 0.28            ; slowly perfused tissue/blood partition coefficient of 3-Methyl-4-nitrophenol

;======================================================================
;Kinetic parameters
;======================================================================
;Fa=Fraction absorbed
Fa = 0.7            ; JMPR 2000
;Absorption/transfer rate constant
kaS=0.32            ; a value set equal to that for diazinon, Zhao et al, 2021 (stomach; /hr)
kaI=0.59            ; a value set equal to that for diazinon, Zhao et al, 2021 (intestine; /hr)
ksI=0.48            ; a value set equal to that for diazinon, Zhao et al, 2021 (transfer stomach-intestine; /hr)
;----------------------------------------------------------------------------------------------------------------
;Metabolism liver
MPL=32  ; scaling factor of human liver microsome (mg microsomal protein /g liver), (Bater et al. 2007)
;based on metabolite formation from FNT to FNO, scaled maximum rate of metabolism (umol/hr)
VMax1c= 1.68 {nmol/min/mg microsomal protein}        ; (FNT-->FNO, CYP450), data derived from experiment
VMax1 = VMax1c/1000*60*MPL*1000*VL                   ; 1000-->nmol to umol
                                                     ; 60-->min to hour
                                                     ; MPL-->mg microsomal protein /g liver
                                                     ; 1000-->kg to g (liver)
                                                     ; VL-->volume of liver tissue {kg} (VLc*BW)
;affinity constants (umol/L)
Km1 = 21.56         ; data derived from experiment
;----------------------------------------------------------------------------------------------------------------
;Metabolism liver
;based on metabolite formation from FNT to MNP, scaled maximum rate of metabolism (umol/hr)
VMax2c = 0.1825 {nmol/min/mg microsomal protein}     ; (FNT-->MNP, CYP450), data derived from experiment
VMax2 = VMax2c/1000*60*MPL*1000*VL                   ; 1000-->nmol to umol
                                                     ; 60-->min to hour
                                                     ; MPL-->mg microsomal protein /g liver
                                                     ; 1000-->kg to g  (liver)
                                                     ; VL-->volume of liver tissue {kg}(VLc*BW)
;affinity constants (umol/L)
Km2 = 4.084         ; data derived from experiment
;----------------------------------------------------------------------------------------------------------------
;Metabolism liver
;based on metabolite formation from FNO to MNP, scaled maximum rate of metabolism (umol/hr)
VMax3c= 3.089 {nmol/min/mg}                          ; (FNO-->MNP, PON1), data derived from experiment
VMax3 = VMax3c/1000*60*MPL*1000*VL                   ; 1000-->nmol to umol
                                                     ; 60-->min to hour
                                                     ; MPL-->mg microsomal protein /g liver
                                                     ; 1000-->kg to g  (liver)
                                                     ; VL-->volume of liver tissue{kg} (VLc*BW)
;affinity constants (umol/L)
Km3 =12564          ; data derived from experiment
;----------------------------------------------------------------------------------------------------------------
;Metabolism blood
MPB=66  ;scaling factor of plasma protein (mg plasma protein / mL plasma-->total protein concentration of plasma), data obtained
from experiment
;based on metabolite formation of from FNO to MNP, scaled maximum rate of metabolism (umol/hr)
VMax4c=2.142 {nmol/min/mg}
VMax4 = VMax4c/1000*60*MPB*1000*VB*0.55              ;1000-->nmol to umol
                                                     ; 60-->min to hour
                                                     ; MPB-->mg plasma protein /mL plasma
                                                     ; 1000-->L to mL  (blood)
                                                     ; VB-->volume of blood {L} (VBc*BW)
                                                     ; 0.55--> plasma makes up about 55% of total blood volume, data
                                                     derived from https:www.blood.co.uk/why-give-blood/how-blood-is-
                                                     used/blood-components/plasma/, since the PON1 is mainly exist in
                                                     plasma, thus we regards metabolism in plasma is equal to metabolism
                                                     in blood.
;affinity constants (umol/L)
Km4 =5489           ; data derived from experiment
;----------------------------------------------------------------------------------------------------------------
;Excretion from kidney via glomerular filtration
;Glomerular filtration
GFR = 1.8                       ; (mL/min/kg bw), Walton et al., 2004
GF = GFR/1000*BW*60             ; (L/hr), human glomerular filtration rate
```

```
; Fraction unbound, predicted by QIVIVE tools (Punt et al. 2021)
FupFNO = 0.158                ; Fraction unbound in plasma (Fup) for FNO
FupliverFNT = 0.643           ; Adjusted Fup for FNT for hepatic metabolism (Poulin and Haddad. 2021)
FupliverFNO = 0.670           ; Adjusted Fup for FNO for hepatic metabolism (Poulin and Haddad. 2021)

;=====================================================================
;Run settings
;=====================================================================
;Molecular weight
MWFNT = 277.23                ; Molecular weight Fenitrothion (FNT)
MWFNO = 261.17                ; Molecular weight Fenitrooxon (FNO)
MWMNP = 153.14                ; Molecular weight 3-Methyl-4-nitrophenol (MNP)

;Oral dose
ODOSEmg = 0.18                                ; given oral dose in mg/kg bw
ODOSEumol2 = ODOSEmg*1E-3/MWFNT*1E6   ; given oral dose recalculated to umol/kg bw
ODOSEumol=ODOSEumol2*BW;                      ; given oral dose in umol

;Time
Starttime = 0        ; in hr
Stoptime = 24        ; in hr

;=====================================================================
;Model calculations
;Fenitrothion (FNT) model
;=====================================================================
;GI-tract compartment
;Ast1  = amount of FNT remaining in stomach (umol)
Ast1'  = -kaS*Ast1-ksI*Ast1
Init Ast1 =ODOSEumol*Fa

;Ast2  = amount of FNT remaining in intestine (umol)
Ast2' =ksI*Ast1-kaI*Ast2
Init Ast2 =0
;---------------------------------------------------------------------------------------------------------------------
;liver compartment
;ALFNT = Amount FNT in liver tissue (umol)
ALFNT' = kaS*Ast1 + kaI*Ast2 + QL*(CBFNT - CVLFNT) - AMFO'- AMFT1'
Init ALFNT = 0
CLFNT = ALFNT/VL
CVLFNT = CLFNT/PLFNT

;AMFO = amount FNT metabolized to the metabolite FNO
AMFO' =VMax1*CVLFNT*(FupliverFNT/0.55)/(Km1 + CVLFNT*(FupliverFNT/0.55))
Init AMFO = 0

;AMFT1 = amount FNT metabolized to the metabolite MNP (and DMTP)
AMFT1' = VMax2*CVLFNT*(FupliverFNT/0.55)/(Km2 + CVLFNT*(FupliverFNT/0.55))
Init AMFT1 = 0
; 0.55 is the BPr value for FNT as a neutral compound, which is used here to correct the adjusted unbound fraction in liver plasma
(Fupliver) to unbound fraction in blood
;---------------------------------------------------------------------------------------------------------------------
;kidney compartment
;AKFNT = Amount FNT in kidney tissue (umol)
AKFNT' = QK*(CBFNT-CVKFNT) - AFNTexe'
Init AKFNT = 0
CKFNT = AKFNT/VK
CVKFNT = CKFNT/PKFNT

;AFNTexe = Amount of FNT eliminated to urine (umol)
AFNTexe' = GF * CVKFNT
Init AFNTexe = 0
;---------------------------------------------------------------------------------------------------------------------
;fat compartment
;AFFNT = Amount FNT in fat tissue (umol)
AFFNT' = QF*(CBFNT-CVFFNT)
Init AFFNT = 0
CFFNT = AFFNT/VF
CVFFNT = CFFNT/PFFNT
;---------------------------------------------------------------------------------------------------------------------
;tissue compartment (rapidly perfused tissue)
```

```
;ARFNT = Amount FNT in rapidly perfused tissue (umol)
ARFNT' = QR*(CBFNT-CVRFNT)
Init ARFNT = 0
CRFNT = ARFNT/VR
CVRFNT = CRFNT/PRFNT
;---------------------------------------------------------------------------------------------------------------
;tissue compartment (slowly perfused tissue)
;ASFNT = Amount FNT in slowly perfused tissue (umol)
ASFNT' = QS*(CBFNT-CVSFNT)
Init ASFNT = 0
CSFNT = ASFNT/VS
CVSFNT = CSFNT/PSFNT
;---------------------------------------------------------------------------------------------------------------
;blood compartment
;ABFNT = Amount FNT in blood (umol)
ABFNT' = QF*CVFFNT +QL*CVLFNT  +QK*CVKFNT +QS*CVSFNT +QR*CVRFNT -(QF+QL+QK+QS+QR)*CBFNT
Init ABFNT = 0
CBFNT = ABFNT/VB

;==========================================================================
;Fenitrooxon (FNO) submodel
;==========================================================================
;liver compartment
;ALFNO = Amount FNO in liver tissue (umol)
ALFNO' =  QL*(CBFNO -CVLFNO)+ AMFO' - AMFT2'
Init ALFNO = 0
CLFNO = ALFNO/VL
CVLFNO = CLFNO/PLFNO

;AMFT2 = Amount FNO metabolized to the metabolite MNP (and DMP)
AMFT2' = VMax3*CVLFNO*(FupliverFNO/0.55)/(Km3 + CVLFNO*(FupliverFNO/0.55))
Init AMFT2  = 0
; 0.55 is the BPr value for FNO as a neutral compound, which is used here to correct the adjusted unbound fraction in liver plasma (Fupliver) to unbound fraction in blood
;---------------------------------------------------------------------------------------------------------------
;kidney compartment
;AKFNO = Amount FNO in kidney tissue (umol)
AKFNO' = QK*(CBFNO-CVKFNO) - AFNOexe'
Init AKFNO = 0
CKFNO = AKFNO/VK
CVKFNO = CKFNO/PKFNO

;AFNOexe = Amount of FNO eliminated to urine (umol)
AFNOexe' = GF * CVKFNO
Init AFNOexe = 0
;---------------------------------------------------------------------------------------------------------------
;fat compartment
;AFFNO = Amount FNO in fat tissue (umol)
AFFNO' = QF*(CBFNO-CVFFNO)
Init AFFNO = 0
CFFNO = AFFNO/VF
CVFFNO = CFFNO/PFFNO
 ;---------------------------------------------------------------------------------------------------------------
;tissue compartment (rapidly perfused tissue)
;ARFNO = Amount FNO in rapidly perfused tissue (umol)
ARFNO' = QR*(CBFNO-CVRFNO)
Init ARFNO = 0
CRFNO = ARFNO/VR
CVRFNO = CRFNO/PRFNO
;---------------------------------------------------------------------------------------------------------------
;tissue compartment (slowly perfused tissue)
;ASFNO = Amount of FNO in slowly perfused tissue (umol)
ASFNO' = QS*(CBFNO-CVSFNO)
Init ASFNO = 0
CSFNO = ASFNO/VS
CVSFNO = CSFNO/PSFNO
;---------------------------------------------------------------------------------------------------------------
;blood compartment
;ABFNO = Amount FNO in blood (umol)
ABFNO' = QF*CVFFNO +QL*CVLFNO  +QK*CVKFNO +QS*CVSFNO +QR*CVRFNO -(QF+QL+QK+QS+QR)*CBFNO - AMFT3'
```

```
Init ABFNO = 0
CBFNO = ABFNO/VB

;AMFT3 = amount FNO metabolized to the metabolite MNP (and DMP)
AMFT3' = VMax4*CBFNO*(FupFNO/0.55)/(Km4 + CBFNO*(FupFNO/0.55))
Init AMFT3  = 0
; 0.55 is the BPr value for FNT as a neutral compound, which is used here to correct the unbound fraction in plasma (Fup) to
unbound fraction in blood

;========================================================================
;3-Methyl-4-nitrophenol (MNP) submodel
;========================================================================
;liver compartment
;ALMNP = Amount MNP in liver tissue (umol)
ALMNP' =  QL*(CBMNP - CVLMNP) +AMFT1' +AMFT2'
Init ALMNP = 0
CLMNP = ALMNP/VL
CVLMNP = CLMNP/PLMNP
;---------------------------------------------------------------------------------------------------------------------
;kidney compartment
;AKMNP = Amount MNP in kidney tissue (umol)
AKMNP' = QK*(CBMNP-CVKMNP) - AMNPexe'
Init AKMNP = 0
CKMNP = AKMNP/VK
CVKMNP = CKMNP/PKMNP

;AMNPexe = Amount MNP eliminated to urine (umol)
AMNPexe' = GF * CVKMNP
Init AMNPexe = 0
;---------------------------------------------------------------------------------------------------------------------
;fat compartment
;AFMNP = Amount MNP in fat tissue (umol)
AFMNP' = QF*(CBMNP-CVFMNP)
Init AFMNP = 0
CFMNP = AFMNP/VF
CVFMNP = CFMNP/PFMNP
 ;---------------------------------------------------------------------------------------------------------------------
;tissue compartment (rapidly perfused tissue)
;ARMNP = Amount MNP in rapidly perfused tissue (umol)
ARMNP' = QR*(CBMNP-CVRMNP)
Init ARMNP = 0
CRMNP = ARMNP/VR
CVRMNP = CRMNP/PRMNP
;---------------------------------------------------------------------------------------------------------------------
;tissue compartment (slowly perfused tissue)
;ASMNP = Amount of MNP in slowly perfused tissue (umol)
ASMNP' = QS*(CBMNP-CVSMNP)
Init ASMNP = 0
CSMNP = ASMNP/VS
CVSMNP = CSMNP/PSMNP
;---------------------------------------------------------------------------------------------------------------------
; blood compartment
;ABMNP = Amount MNP in blood (umol)
ABMNP' = QF*CVFMNP +QL*CVLMNP  +QK*CVKMNP +QS*CVSMNP +QR*CVRMNP +AMFT3'-
(QF+QL+QK+QS+QR)*CBMNP
Init ABMNP = 0
CBMNP = ABMNP/VB

;========================================================================
;Mass balance calculations for FNT model
TotalFNT = ODOSEumol *Fa
CalculatedFNT = Ast1 +Ast2 +ALFNT +AKFNT +AFFNT +ASFNT +ARFNT +ABFNT+AMFO +AMFT1 +AFNTexe
ERRORFNT=(TotalFNT-CalculatedFNT)/(TotalFNT+1E-30)*100
MASSBBALFNT=TotalFNT-CalculatedFNT + 1

;========================================================================
;Mass balance calculations for FNO sub-model
TotalFNO = AMFO
CalculatedFNO = ALFNO +AKFNO +AFFNO +ASFNO +ARFNO +ABFNO +AMFT2 +AMFT3 +AFNOexe
ERRORFNO=(TotalFNO-CalculatedFNO)/(TotalFNO+1E-30)*100
MASSBBALFNO=TotalFNO-CalculatedFNO + 1
```

```
;=========================================================================
;Mass balance calculations for MNP sub-model
TotalMNP = AMFT1 +AMFT2 +AMFT3
CalculatedMNP = ALMNP +AKMNP +AFMNP +ASMNP +ARMNP +ABMNP +AMNPexe
ERRORMNP=(TotalMNP-CalculatedMNP)/(TotalMNP+1E-30)*100
MASSBBALMNP=TotalMNP-CalculatedMNP + 1
```

References

APVMA (2023). Acute reference doses (ARfD) for agricultural and veterinary chemicals used in food producing crops or animals (Edition 2/2023). https://apvma.gov.au/node/98336 (Accessed August 27, 2023).

Badhan RKS, Chenel M, Penny JI (2014). Development of a physiologically-based pharmacokinetic model of the rat central nervous system. Pharmaceutics 6:97-136.

Barter ZE, Bayliss MK, Beaune PH, Boobis AR, Carlile DJ, Edwards RJ, Houston JB, Lake BG, Lipscomb JC, Pelkonen OR, Tucker GT, Rostami-Hodjegan A (2007). Scaling factors for the extrapolation of *in vivo* metabolic drug clearance from *in vitro* data: reaching a consensus on values of human microsomal protein and hepatocellularity per gram of liver. Current Drug Metabolism 8:33-45.

Brown RP, Delp MD, Lindstedt SL, Rhomberg LR, Beliles RP (1997). Physiological parameter values for physiologically based pharmacokinetic models. Toxicology and Industrial Health 13:407-484.

Chemicalize: https://chemicalize.com/welcome (Accessed October 18, 2023).

EFSA (2006). Conclusion regarding the peer review of the pesticide risk assessment of the active substance fenitrothion. EFSA Scientific Report 59:1-80.

Gearhart JM, Jepson GW, Clewell HJ, Andersen ME, Conolly RB (1990). Physiologically based pharmacokinetic and pharmacodynamic model for the inhibition of acetylcholinesterase by diisopropylfluorophosphate. Toxicology and Applied Pharmacology 106:295-310.

Hladká A, Nosáľ M (1967). The determination of the exposition to metathion (fenitrotion) on the basis of excreting its metabolite p-nitro-m-cresol through urine in rats. Int. Archiv für Gewerbepathologie und Gewerbehygiene 23:209-214.

JMPR (2000). https://www.fao.org/3/cb2757en/cb2757en.pdf (Accessed April 28, 2023).

JMPR (2003). Unpublished data of Schepler, K.; Schick, M. (2002): Partition coefficient (n-octanol:water) of ^{14}C-fenitrothion. https://www.fao.org/fileadmin/templates/agphome/documents/Pests_Pesticides/JMPR/Evaluation03/fenitrothion_2003.pdf (Accessed October 18, 2023).

Kasteel EEJ, Nijmeijer SM, Darney K, Lautz LS, Dorne JLCM, Kramer NI, Westerink RHS (2020). Acetylcholinesterase inhibition in electric eel and human donor blood: An in vitro approach to investigate interspecies differences and human variability in toxicodynamics. Arch. Toxicol. 94, 4055−4065.

Meaklim J, Yang J, Drummer OH, Killalea S, Staikos V, Horomidis S, Rutherford D, Ioannides-Demos LL, Lim S, McLean AJ, McNeil JJ (2003). Fenitrothion: Toxicokinetics and toxicologic evaluation in human volunteers. Environmental Health Perspectives 111:305-308.

Medinsky MA, Leavens TL, Csanády GA, Gargas ML, Bond JA (1994). *In vivo* metabolism of butadiene by mice and rats: A comparison of physiological model predictions and experimental data. Carcinogenesis 15:1329-1340.

Miyamoto J, Mihara K, Hosokawa S (1976). Comparative metabolism of m-methyl-^{14}C-Sumithion in several species of mammals *in vivo*. Journal of Pesticide Science 1:9-21.

Miyamoto J, Mihara K, Kadota T, Okuno Y (1977). Toxicity and metabolism *in vivo* of fenitrothion in rats with experimental hepatic lesion. Journal of Pesticide Science 2:271-277.

Nosáľ M, Hladká A (1968). Determination of the exposure to fenitrothion (O,O-dimethyl-O/3-methyl-4-nitrophenyl/thiophosphate) on the basis of the excretion of p-nitro-m-cresol by the urine of the persons tested. Int Arch Gewerbepath Gewerbehyg 25:28-38.

OECD (1994). SIDS initial assessment report for 3-methyl-4-nitrophenol. https://hpvchemicals.oecd.org/UI/handler.axd?id=7816cee9-8d4c-468c-9d3e-54b4e715d759 (Accessed October 18, 2023).

Pan X, Wang H, Li C, Zhang JZH, Ji C (2021). MolGpka: A web server for small molecule pKa prediction using a graph-convolutional neural network. Journal of Chemical Information and Modeling 61(7):3159-3165.

Pehkonen SO, Zhang Q (2002). The degradation of organophosphorus pesticides in natural waters: A critical review. Critical Reviews in Environmental Science and Technology 32(1):17-72.

Pires DEV, Blundell TL, Ascher DB (2015). pkCSM: Predicting small-molecule pharmacokinetic and toxicity properties using graph-based signatures. J. Med. Chem. 58:4066-4072.

Poulin P, Haddad S (2021). A new guidance for the prediction of hepatic clearance in the early drug discovery and development from the *in vitro*-to-*in vivo* extrapolation method and an approach for exploring whether an albumin-mediated hepatic uptake phenomenon could be presentunder in vivo conditions. Journal of Pharmaceutical Sciences 110:2841-2858.

PubChem: https://pubchem.ncbi.nlm.nih.gov/ (Accessed October 18, 2023).

Punt A, Pinckaers N, Peijnenburg A, Louisse J (2021). Development of a web-based toolbox to support Quantitative *In-Vitro*-to-*In-Vivo* Extrapolations (QIVIVE) within nonanimal testing strategies. Chemical Research in Toxicology 34:460-472.

Shahpoury P, Kitanovski Z, Lammel G (2018). Snow scavenging and phase partitioning of nitrated and oxygenated aromatic hydrocarbons in polluted and remote environments in central Europe and the European Arctic. Atmos. Chem. Phys., 18:13495-13510.

Story P, Vanek M, Mueller J, Hawker D (2012). Applications of Equilibrium Passive Samplers to Monitor Pesticides in Water Bodies During a Locust Control Event – Quilpie 2002. Australian Plague Locust Commission Research Report Environment Program. https://www.agriculture.gov.au/sites/default/files/sitecollectiondocuments/animal-plant/aplc/research-papers/passive-sampler-report.pdf (Accessed October 18, 2023).

Thermo Fisher Scientific (2020). User guide: Pierce BCA protein assay kit. https://www.thermofisher.com/document-connect/document-connect.html?url=https%3A%2F%2Fassets.thermofisher.com%2FTFS-Assets%2FLSG%2Fmanuals%2FMAN0011430_Pierce_BCA_Protein_Asy_UG.pdf&title=VXNlciBHdWlkZTogUGllcmNlIEJDQSBQcm90ZWluIEFzc2F5IEtpdA== (Accessed August 27, 2023).

US EPA (2010). https://www3.epa.gov/pesticides/chem_search/cleared_reviews/csr_PC-105901_10-Nov-10.pdf (Accessed August 27, 2023).

Walton K, Dorne JLCM, Renwick AG (2004). Species-specific uncertainty factors for compounds eliminated principally by renal excretion in humans. Food and Chemical Toxicology 42(2):261-274.

Wang D, Schramm V, Pool J, Pardali E, Brandenburg A, Rietjens IMCM, Boogaard PJ (2022). The effect of alkyl substitution on the oxidative metabolism and mutagenicity of phenanthrene. Arch. Toxicol. 96, 1109-1131.

WHO (2004). Fenitrothion in Drinking-water. Background document for development of WHO Guidelines for Drinking-water Quality. https://cdn.who.int/media/docs/default-source/wash-documents/wash-chemicals/fenitrothion-bd.pdf?sfvrsn=c8504e7c_4 (Accessed October 18, 2023).

WHO (2010). Characterization and application of physiologically based pharmacokinetic models in risk assessment. Geneva, Switzerland.

Zhao S, Kamelia L, Boonpawa R, Wesseling S, Spenkelink B, Rietjens IMCM (2019). Physiologically based kinetic modeling-facilitated reverse dosimetry to predict in vivo red blood cell acetylcholinesterase inhibition following exposure to chlorpyrifos in the Caucasian and Chinese population. Toxicol. Sci. 171(1), 69-83.

Zhao S, Wesseling S, Spenkelink B, Rietjens IMCM (2021). Physiologically based kinetic modelling based prediction of *in vivo* rat and human acetylcholinesterase (AChE) inhibition upon exposure to diazinon. Archives of Toxicology 95:1573-1593.
